# Ultrasonic and mechanochemical strategies for the synthesis of oxindole scaffolds mediated by hypervalent iodine(iii) reagents

**DOI:** 10.1039/d6ra05034d

**Published:** 2026-07-06

**Authors:** Niharan Sivaraj, Fateh V. Singh

**Affiliations:** a Department of Chemistry, School of Advanced Sciences, Vellore Institute of Technology – Chennai Campus Chennai – 600127 Tamil Nadu India Fatehveer.singh@vit.ac.in

## Abstract

We report a rapid and efficient methodology for the synthesis of 6*H*-isoindolo[2,1-*a*] indol-6-one scaffolds 18a–k mediated by hypervalent iodine(iii) reagents using ultrasonic and mechanochemical activation strategies. These non-conventional approaches significantly enhance reaction rates and reduce reaction times compared to conventional methods. This protocol employs readily accessible starting materials under mild conditions to afford structurally diverse oxindole derivatives in good to excellent yields (60–93%) with broad functional group tolerance. Ultrasonic irradiation facilitates rapid molecular interactions *via* acoustic cavitation, while mechanochemical activation under liquid-assisted grinding (LAG) conditions enables efficient transformation using only a small amount of solvent. This approach demonstrates strong operational robustness across a range of substrates and offers a practical alternative to conventional solution-phase synthesis. The hypervalent iodine(iii) reagent serves as an effective oxidant and cyclization promoter, enabling selective formation of the oxindole core. This study demonstrates the effectiveness of integrating ultrasonic and mechanochemical activation techniques with hypervalent iodine chemistry for the non-conventional activation strategy for the construction of functionalized heterocycles.

## Abstract 2

A rapid and efficient synthesis of 6*H*-isoindolo[2,1-*a*] indol-6-one derivatives 18 was achieved through hypervalent iodine(iii)-mediated oxidative cyclization under ultrasonic and mechanochemical activation.

## Graphical abstract

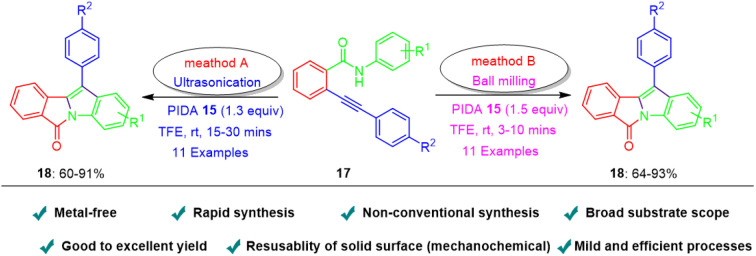

## Introduction

**Fig. 1:**
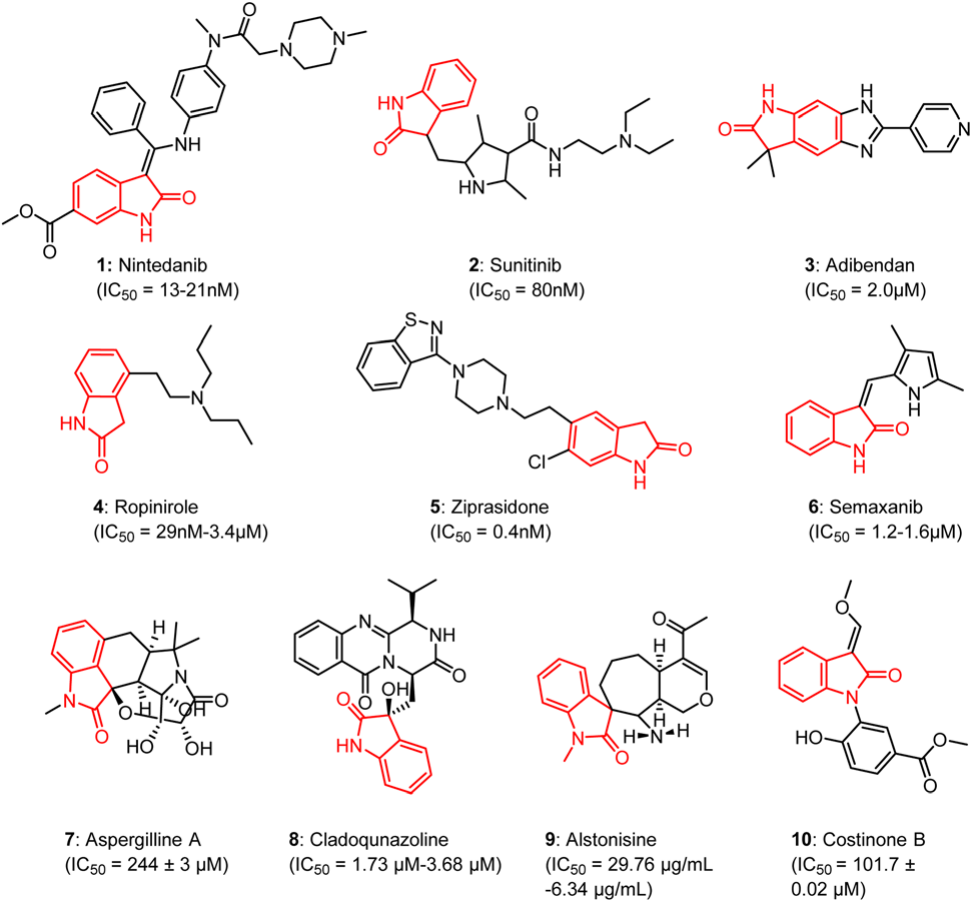
Structure of commercially and natural available drugs with an oxindole core.

Oxindole is a privileged heterocycle that occurs throughout nature and in many biologically active compounds.^1–3^ especially present in the bodily fluids and tissue of mammals and as natural products of numerous plants.^4–6^ Structurally, oxindole is a compound consisting of a benzene ring and a five-membered lactam structure, thus providing the molecule with rigidity and planarity and making it susceptible to structural modification. Due to its special molecular structure, which features both a carbonyl group and a nitrogen atom, this compound is capable of participating in intermolecular interactions such as hydrogen bonding and π–π stacking.^7^ These intermolecular interactions are responsible for the biological activity of the molecule.^8^ Oxindole analogues have proven to be useful in medicinal chemistry^9^ because they exhibit numerous pharmacological effects such as anticancer,^10,11^ antiviral,^12,13^ anti-inflammatory^14,15^ and antimicrobial activity.^16,17^ Several drugs that inhibit the activity of kinases are known derivatives of oxindole.^18^ Several oxindole derivatives, both synthetic and naturally occurring, underscore the broad pharmacological relevance of this scaffold. Clinically important drugs such as Nintedanib^19^**1** and Sunitinib^20^**2** demonstrate potent kinase inhibitory activity, while Ropinirole^21^**4** and Ziprasidone^22^ 5 are widely used in treating neurological disorders; other derivatives like Adibendan^23^**3** exhibit significant cardiovascular effects, although Semaxanib^24^**6** failed in late-stage trials (Fig. 1).

In addition to synthetic drugs, several natural oxindole alkaloids have been identified, including Aspergilline A^25^ 7 from Aspergillus versicolor, Cladoquinazoline^26,27^ 8 from Cladosporium species with anti-influenza potential and Alstonisine^28,29^ 9 isolated from Alstonia muelleriana. Furthermore, Costinone B^30^**10** obtained from Isatis costata exhibits inhibitory activity against lipoxygenases and butyrylcholinesterase (Fig. 1), where structural features such as *N*-aryl substitution and C-3 oxygenation play a crucial role, highlighting the importance of substitution patterns in modulating biological activity.

The functionalization of oxindole at the third carbon is one of the ways to prepare structurally distinct molecules with desired biological properties. It is widely known that oxindoles have readily two tautomerizing hydroxyl isomers.^31^ The development of efficient and selective methodologies for constructing substituted oxindoles remains an active area of research, driven by the demand for novel compounds with improved activity and selectivity.

### Synthesis of oxindoles

**Scheme 1:**
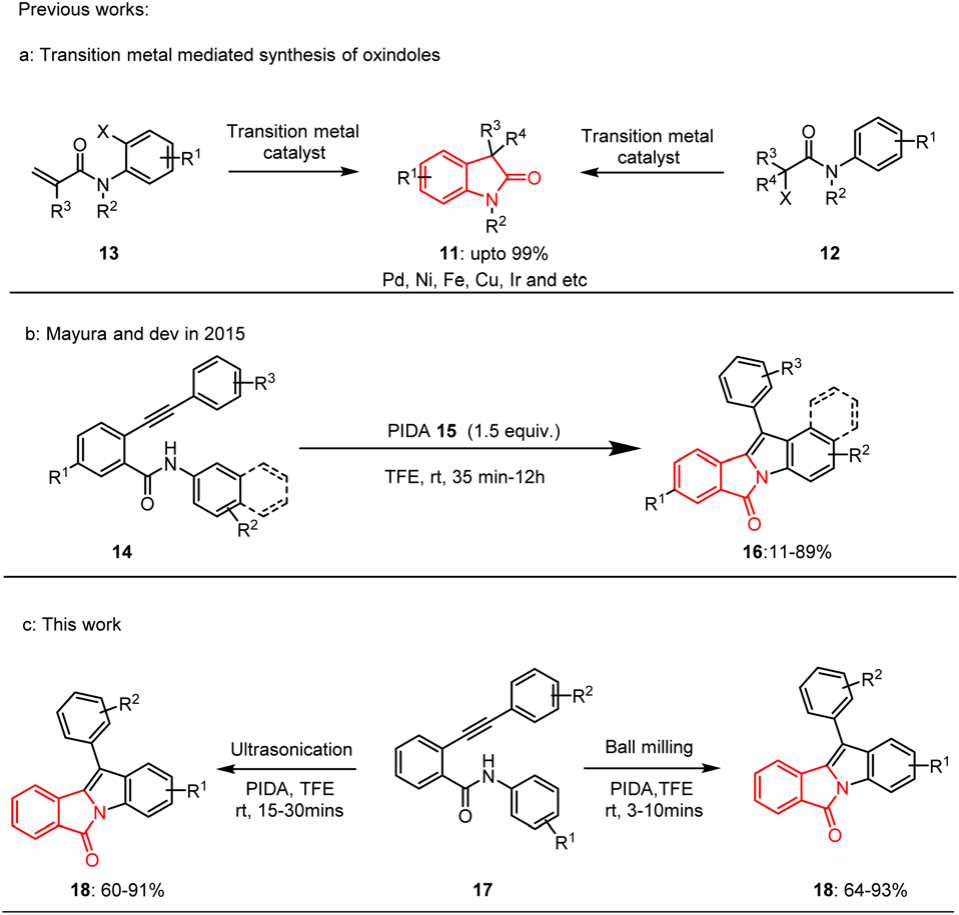
Schematic representation of various synthetic protocol of oxindoles.

In the literature, early approaches to oxindole synthesis primarily involved the reduction of isatins or intramolecular Friedel–Crafts acylation of aniline-derived α-haloamides, which laid the foundation for constructing the oxindole core.^32^ Subsequently, most established methodologies have relied on prefunctionalized oxindole scaffolds, particularly for accessing 3,3′-disubstituted oxindoles through conventional derivatization strategies at the C-3 position.^33^ Over time, a wide array of synthetic routes has been developed for assembling the oxindole framework, among which transition metal-mediated oxidative C–H and N–H cyclizations have emerged as powerful and extensively utilized strategies, employing catalysts based on palladium, copper, rhodium and ruthenium to enable direct annulation under oxidative conditions (Scheme 1a).^34^

To overcome the limitations associated with the a forementioned methods, transition metal-free techniques have been established for the synthesis of oxindole derivatives.^35,36^ Nevertheless, these protocols still rely on the use of strong oxidizing agents such as elemental iodine.^37,38^ Because of their low toxicity, ease of handling, strong oxidizing ability and environmental friendliness, hypervalent iodine(iii) reagents have emerged as appealing alternatives to traditional metal catalysts. Under mild circumstances, phenyliodine diacetate (PIDA) accelerated the intramolecular oxidative annulation process resulting in the required oxindole framework. According to previous publications by Mayura and Dev, treating the alkynyl amide substrate with PIDA (1.5 equiv) in trifluoroethanol at room temperature resulted in the cyclized oxindole derivatives by oxidative cyclization followed by intramolecular rearrangement (Scheme 1b).^39^ Although the standard approach produced the desired products in moderate to good yields, the reaction typically required extended reaction periods ranging from 35 minutes to 12 hours under continuous stirring conditions. Furthermore, the old solution-phase technology required significant solvent usage and higher energy consumption, which restricts its sustainability from a green chemistry standpoint. The development of alternative activation strategies that improve reaction efficiency and operational practicality remains of considerable interest.

To improve the efficiency of the transformation and reduce the environmental burden associated with conventional methods, non-conventional synthetic approaches such as ultrasonication and mechanochemical ball milling were explored.^8^ These activation techniques have recently attracted considerable interest because of their ability to accelerate reactions under fast and mild conditions (Scheme 1c).

Motivated by the biological significance of oxindole-containing scaffolds and the growing demand for efficient synthetic techniques, we attempted to create an efficient hypervalent iodine-mediated cyclization strategy under ultrasonic and mechanochemical activation conditions. The exhibited approach produces structurally diverse 6*H*-isoindolo[2,1-*a*]indol-6-one derivatives in good to excellent yields with broad functional group tolerance, operational simplicity and convenient product isolation. Furthermore, non-conventional activation strategies dramatically shorten reaction durations and enhance reaction rate resulting in a operationally simple approach to fused oxindole framework synthesis (Table 1).

**Table 1:** Comparative evaluation of conventional, ultrasonic and mechanochemical approaches for the synthesis of 6*H*-isoindolo[2,1-*a*] indol-6-one derivatives 18

Method	Yield %	Time	Solvent	Condition
Maurya *et al.*^39^	89	35 min to 12 h	TFE (1.5 mL)	Conventional stirring
Ultrasonication (this work)	60–91	15–30 min	TFE (1.0 mL)	US
Ball milling (this work)	64–93	3–10 min	TFE (1.0 mL)	LAG

## Results and discussion

**Scheme 2:**
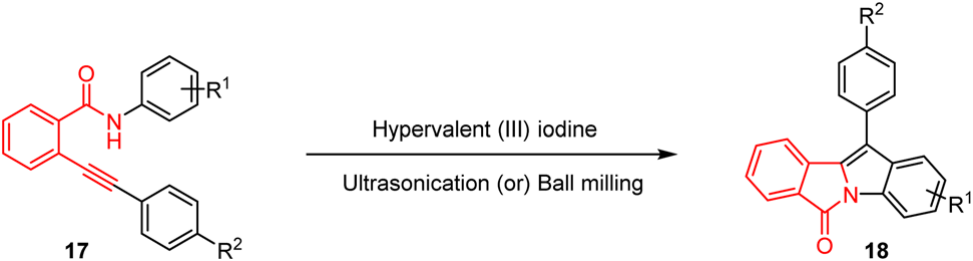
Hypervalent (III) iodine reagent mediated-cyclization of *N*-phenyl-2-(1-arylethynyl) benzamides 17 into 11-aryl-6*H*-isoindolo[2,1-*a*]-indol-6-ones 18.

In this study, we proposed the synthetic route for the synthesis of 6*H*-isoindolo[2,1-*a*]indol-6-one compounds 18 employing hypervalent iodine reagent as an oxidant under mild reaction conditions (Scheme 2). Hypervalent iodine(iii) reagent was employed to cyclize *N*-phenyl-2-(1-arylethynyl) benzamides 17 into 11-aryl-6*H*-isoindolo[2,1-*a*]-indol-6-ones 18.

**Scheme 3:**
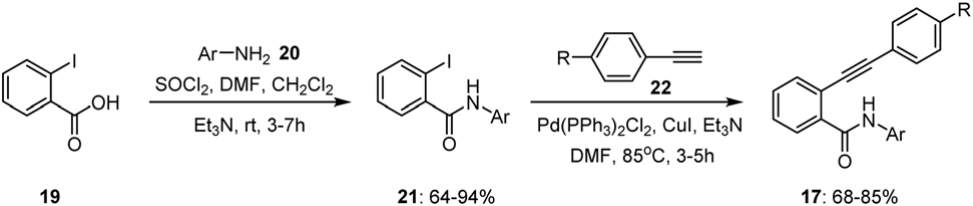
Approach for Synthesizing *N*-phenyl-2-(1-arylethynyl) benzamides 17.

Initially, benzamide derivatives 21 were synthesized from 2-iodobenzoic acid 19 through a two-step procedure. Treatment of 2-iodobenzoic acid 19 with thionyl chloride generated the corresponding benzoyl chloride intermediate, which subsequently underwent coupling with aromatic amines 20 in the presence of Et_3_N as a base to afford the desired benzamides 21 in good to excellent yields (Scheme 3).^40^

Subsequently, *N*-phenyl-2-(1-arylethynyl) benzamides 17 were then produced by the Sonogashira cross-coupling reaction between phenylacetylene 22 and 2-iodobenzamides 21. The intended *N*-phenyl-2-(1-arylethynyl) benzamides 17 were effectively produced in good yields when the reaction was conducted at 85 °C with a palladium catalyst present in an inert environment (Scheme 3).^41^ After that, we worked to determine the ideal reaction conditions for the cyclization of *N*-phenyl-2-(1-arylethynyl) benzamides 17. As a model substrate, *N*-phenyl-2-(phenylethynyl) benzamide 17a was chosen.

A number of experiments were conducted using substrate 17a under ultrasonication (US) and mechanochemical ball-milling (BM) conditions with various oxidants and varying equivalents of oxidants in order to determine the ideal reaction conditions for the synthesis of fused oxindole derivative 18a (Table 2). Initially, Iodobenzene (PhI, 1.5 equiv) was tested as an oxidant under ultrasonication and ball-milling conditions for 60 minutes; however, no formation of the intended product 18a was detected (Table 2, Entry 1 and Entry 10). These findings demonstrate that iodobenzene alone is incapable of stimulating the cyclization reaction, underlining the need of hypervalent iodine(iii) species in the oxidative cyclization process. The hypervalent iodine reagent PIDA was then explored due to its well-known oxidative capabilities and efficiency in intramolecular cyclization processes. Fortunately, when 1.0 equiv of PIDA was used, the desired oxindole product 18a was achieved in 51% yield under ultrasonication and 55% yield under ball-milling conditions within 5 minutes (Table 2, Entry 2 and Entry 11).

**Table 2:** Optimization of Iodine based oxidant for the cyclization of *N*-phenyl-2-(phenylethynyl) benzamide 17a

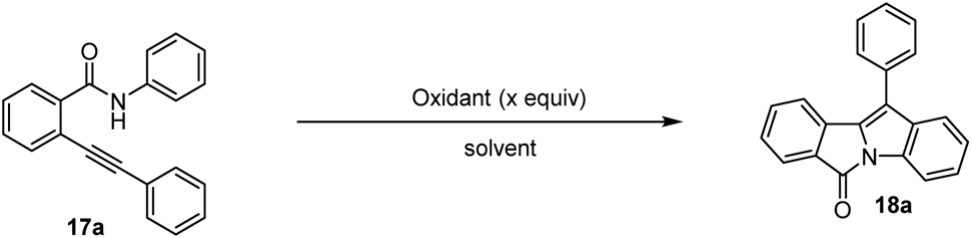			
Entry	Oxidant (equiv)	Reaction time (min)	Yield %
1	PhI (1.5)^a^	60	0
2	PIDA (1.0)^a^	5	51
3	PIDA (1.2)^a^	10	63
4	PIDA (1.3)^a^	10	71
5	**PIDA (1.3)** a	15	**89**
6	PIDA (1.5)^a^	15	88
7	PIDA (1.7)^a^	15	89
8	IBX (1.3)^a^	60	0
9	IBX (1.5)^a^	60	0
10	PhI (1.5)^b^	60	0
11	PIDA (1.0)^b^	5	55
12	PIDA (1.2)^b^	5	65
13	PIDA (1.3)^b^	5	74
14	PIDA (1.3)^b^	10	86
15	**PIDA (1.5)** b	**>5**	**89**
16	PIDA (1.7)^b^	>5	89
17	IBX (1.3)^b^	60	0
18	IBX (1.7)^b^	60	0

a Condition a: ultrasonication, oxidant, TFE, rt.

b Condition b: ball milling, oxidant, TFE, rt.

Encouraged by this finding, the amount of PIDA was steadily raised in order to improve reaction efficiency. Increasing the PIDA loading to 1.2 equiv. increased the product yield to 63% under ultrasonication and 65% under ball milling (Table 2, Entry 3 and Entry 12). Further increase was seen with 1.3 equiv. of PIDA, resulting in 71% and 74% yields under ultrasonication and mechanochemical conditions respectively, within short response times (Table 2, Entry 4 and Entry 13). Interestingly, increasing the reaction time under ultrasonication to 15 minutes with 1.3 equiv of PIDA resulted in a considerable increase in product yield with 18a yielding 89% (Table 2, Entry 5). Under ball-milling settings, the reaction was completed in 10 minutes with an 86% of yield (Table 2, Entry 14), indicating the amazing efficiency of mechanochemical activation. Increasing the oxidant loading to 1.5 equiv did not result in a significant improvement under ultrasonication (Table 2, Entry 6), but the ball-milling method produced the product in 89% yield (Table 2, Entry 15). Similarly, using 1.7 equiv. of PIDA did not improve product formation in either condition (Table 2, Entry 7 and Entry 16). To compare the efficacy of different hypervalent iodine oxidants, IBX was tested using both activation methods. However, no desirable product formation was seen even after 60 minutes of adding either 1.3 or 1.5 equiv of IBX (Table 2, Entries 8,9 and Entries 17,18), This could be attributable to its decreased propensity to facilitate electrophilic activation of the alkyne molecule, which is a crucial step in the cyclization process. PIDA, on the other hand, has been shown to effectively assist such conversions by producing reactive iodine(iii) species and it is capable of activating C–C multiple bonds toward intramolecular cyclization. Furthermore, the poor solubility of IBX in TFE may further decrease its efficiency under the reaction conditions.

Overall, the optimization studies determined that PIDA is the most effective oxidant for the current transformation. Among the conditions tested, ultrasonication with 1.3 equiv of PIDA for 15 min and ball milling with 1.5 equiv of PIDA for less than 5 min were found to be the most effective, producing the required fused oxindole derivative 18a in high quantities. Notably, the mechanochemical ball-milling process outperformed ultrasonication in terms of reaction efficiency while requiring much less time.

The preliminary oxidant optimization investigations were initially carried out utilizing TFE as the reaction solvent based on previous studies. Following the identification of PIDA as the best oxidant, additional studies were conducted to investigate the influence of various solvents on reaction efficiency under ultrasonication and ball-milling conditions (Table 2). Since solvent polarity and reactive intermediate stability can greatly influence hypervalent iodine-mediated transformations, numerous commonly used organic solvents were evaluated to discover the best suited reaction medium.

Initially, methanol (MeOH) was tested under optimal oxidant conditions with PIDA (1.3/1.5 equiv). Despite a protracted reaction time of less than 60 minutes, the intended fused oxindole product 18a was only produced in 20% yield (Table 3, Entry 1). This observation prompted the evaluation of many aprotic solvents, including MeCN, DCM, THF, DMSO and CHCl_3_ (Table 3, Entries 2–6). However, no product formation was seen in any of these solvents, and the starting material remained mostly unreacted even after 60 minutes of reaction time. The low reactivity of these solvents can be related to their failure to adequately stabilize the cationic intermediates produced during hypervalent iodine-mediated oxidative cyclization. In contrast, highly fluorinated alcohol solvents have been shown to improve the electrophilic activation ability of hypervalent iodine reagents and facilitate intramolecular cyclization processes *via* strong hydrogen-bonding interactions and ionic intermediate stabilization. Nonetheless, when trifluoroethanol (TFE) was used as the solvent, the reaction proceeded very efficiently under both ultrasonication and ball-milling conditions, producing the desired product 18a in an outstanding 89% yield in less than 5 minutes (Table 3, Entry 7). Its outstanding performance is due to its strong polarity, hydrogen-bond donating ability and excellent capacity to stabilize reactive intermediates involved in the oxidative annulation pathway. Although TFE demonstrated the best reaction performance, the use of fluorinated solvents remains a consideration in the overall assessment of the methodology. Overall, the solvent screening investigations clearly revealed that TFE is required for the oxidative cyclization procedure to proceed successfully.

**Table 3:** Screening of various solvents for the cyclization of *N*-phenyl-2-(phenylethynyl) benzamide 17a

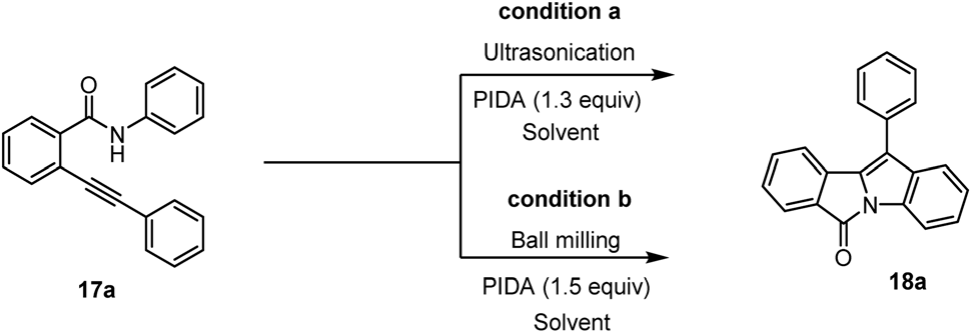				
Entry	Oxidant (equiv)	Solvent	Time (min)	Yield %
1	PIDA (1.3)^a^ or (1.5)^b^	MeOH	<60	20
2	PIDA (1.3)^a^ or (1.5)^b^	MeCN	<60	0
3	PIDA (1.3)^a^ or (1.5)^b^	DCM	<60	0
4	PIDA (1.3)^a^ or (1.5)^b^	THF	<60	0
5	PIDA (1.3)^a^ or (1.5)^b^	DMSO	<60	0
6	PIDA (1.3)^a^ or (1.5)^b^	CHCl_3_	<60	0
**7**	**PIDA (1.3)** a **or (1.5)** b	**TFE**	**>5**	**89**

a All-optimization experiments were performed using 17a (500 mg, 1.683 mmol, 1.0 equiv), PIDA (1.3 equiv) and TFE (1.0 mL) under the indicated reaction conditions.

b Ball milling, PIDA (1.5 equiv), TFE (1.0 mL) under the indicated reaction condition b.

After the completion of optimization studies, a series of oxindole compounds 18a–k were synthesized by the cyclization of *N*-phenyl-2-(1-arylethynyl) benzamides 17a–k using (1.3/1.5 equiv) of PIDA in TFE medium at room temperature. Various electron-donating (EDG) and withdrawing substituents (EWG) on aryl ring of substrate 17 were successfully tolerated, but oxindole products 18 were obtained in higher yield with substrates having electron-donating substituents (Table 4, Entries 8–10). Additionally, the cyclization reaction was also working with substrate 17b having functional group at *ortho* position of aromatic ring although cyclic product 18b was isolated in lower yield. Although fluorine exerts minimal steric demand, the reduced efficiency may also associate with the positional influence of the ortho substituent on the conformational preferences of the substrate and the electronic distribution within the reaction intermediate, which can adversely affect the cyclization process (Table 4, Entry 2).

**Table 4:** The scope of cyclization reaction of *N*-phenyl-2-(1-arylethynyl) benzamides 17a–k to 11-aryl-6*H*-isoindolo[2,1-*a*]-indol-6-ones 18a–k using PIDA 15 as oxidant

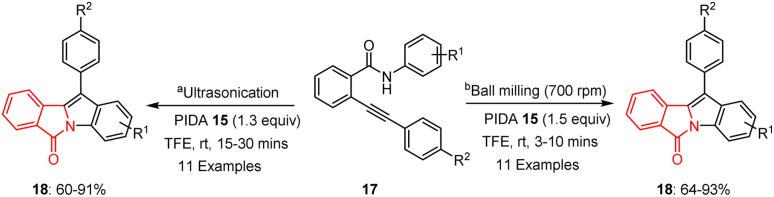						
Entry	R^1^	R^2^	Time (min)		Yield %	
			US	BM	US	BM
1	H	H	15	3	89	89
2	2-Fluoro	H	30	10	60	64
3	4-Fluoro	H	17	8	78	82
4	3-Chloro	H	20	8	70	72
5	4-Chloro	H	25	8	80	84
6	4-Bromo	H	18	10	83	85
7	4-Cyano	H	20	8	81	87
8	4-Methyl	H	20	10	87	91
9	4-Methoxy	H	15	5	91	93
10	4-Butyl	H	25	8	88	89
11	4-Methoxy	4-Butyl	10	5	82	87

**Scheme 4:**
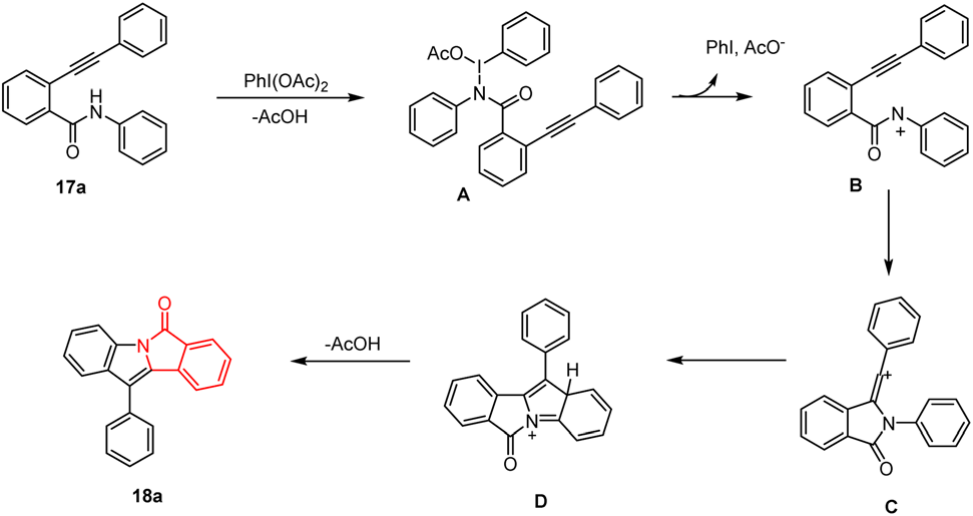
Plausible reaction mechanism for iodine(iii)– mediated cyclization of *N*-phenyl-2-(1-arylethynyl) benzamides 17a to 11-aryl-6*H*-isoindolo[2,1-*a*]-indol-6-ones 18a.

The proposed mechanism for hypervalent iodine-mediated oxidative cyclisation begins with the activation of substrate 17a by PIDA 15 to produce intermediate A, the amide nitrogen interacts with the iodine(iii) site, followed by the removal of acetic acid. The electrophilic nitrenium ion intermediate B is produced by the subsequent reductive elimination of iodobenzene and acetate ions. The activated alkyne moiety then undergoes intramolecular cyclization to generate cyclic intermediate C, which further participates in electrophilic aromatic annulation with the adjacent phenyl ring to yield fused intermediate D. Finally, deprotonation and elimination of acetic acid restore aromaticity, resulting in the synthesis of the desired fused oxindole product 18a (Scheme 4). Under metal-free circumstances, the total transformation proceeds sequentially through nitrogen activation, intramolecular alkyne cyclization and oxidative annulation mediated by the hypervalent iodine reagent.^39,42–44^

## Conclusion

In conclusion, an effective hypervalent iodine-mediated approach for the synthesis of fused oxindole derivatives was devised in the absence of transition metals.^45–53^ The reaction proceeded successfully under ultrasonication and mechanochemical ball-milling conditions with PIDA as the oxidant, yielding the required products in excellent yields in a short reaction time. The established approach has a broad substrate scope and strong functional group tolerance, allowing for both electron-donating and electron-withdrawing substituents on aromatic ring systems. The current approach offers an effective pathway to structurally varied compounds of possible pharmacological interest, given the proven significance of oxindole-containing frameworks in medicinal chemistry. Furthermore, the protocol displayed remarkable performance under non-traditional activation techniques, notably mechanochemical circumstances with shorter reaction times and enhanced reaction rate. The methodology has several advantages including operational simplicity, mild reaction conditions, an environmentally benign reaction profile and increased sustainability demonstrating the potential of hypervalent iodine chemistry combined with non-conventional synthetic approaches in organic synthesis. Furthermore, the successful combination of ultrasonic and mechanochemical activation with hypervalent iodine chemistry provides a practical and efficient approach to process chemistry, enabling shorter reaction times with high efficiency and greener synthesis protocols.

## Author contributions

Niharan Sivaraj: methodology, characterization and writing – original draft; Fateh V Singh: conceptualization, project administration, supervision, writing – review and editing.

## Conflicts of interest

There are no conflicts to declare.

## Supplementary Material

RA-OLF-D6RA05034D-s001

## Data Availability

The synthesis procedure, all the spectroscopic data are given in the supporting information (SI) including the NMR spectra of all the synthesized compounds. Supplementary information is available. See DOI: https://doi.org/10.1039/d6ra05034d.
