# Supplementary material for: Ultrasonic and mechanochemical strategies for the synthesis of oxindole scaffolds mediated by hypervalent iodine(iii) reagents
Source: RSC Adv. 2026 Jul 6;16(36):37700–5. doi: 10.1039/d6ra05034d (PMC13334527; doi:10.1039/d6ra05034d)

Supplementary Information (SI) for RSC Advances.
This journal is © The Royal Society of Chemistry 2026

Supporting Information

Ultrasonic and mechanochemical strategies for the synthesis of oxindole scaffolds mediated by hypervalent iodine (III) reagents

Niharan Sivaraj and Fateh V. Singh*

Chemistry department, SAS, Vellore Institute of Technology - Chennai campus, vandalur-kelambakkam Road, Chennai-600127, Tamil Nadu, India

Phone: +91 44 3993 1472; E-mail: fatehveer.singh@vit.ac.in

Contents	Page No
General information	2
General synthetic procedure and characterisation of 2-iodobenzamides **21a-j**	2 - 4
General synthetic procedure and characterisation of 2-(1-arylethynyl) benzamides **17a-k**	4 - 6
General synthetic procedure for 6*H*-isoindolo[2,1-a] indol-6-ones **18a-k**	7 – 9
^{1}H and ^{13}C NMR for 6*H*-isoindolo[2,1-a] indol-6-ones **18a-k**	10 - 31

1. General Information

The melting points (mp) were determined using a REMI-DDMS (2545) melting point apparatus. A Nicolet Nexus 470FT-IR spectrometer from Thermo Scientific was used to measure the IR spectra with band positions given in reciprocal centimetres. KBr was used to create pellet samples, which were then recorded. Using the specified solvents, ^{1}H and ^{13}C NMR spectra were recorded at 400 MHz and 100 MHz, respectively, using a Bruker AV-400. Under the circumstances of electron ionization (EI), mass spectra (m/z) were acquired. Ultrasonication bath with 33KHz with 100W power and Ball milling instrument (FRITSCH Pulverisette planetary ball milling with jar size of 10ml and agate balls (13 balls)) were used. All the reactions were monitored using thin-layer chromatography (TLC) on pre-coated silica gel sheets and column chromatography was carried out with silica gel 60-120 mesh, which were brought from Hyma Synthesis Pvt. Ltd. Hexane and ethyl acetate were worked as mobile phase and were procured from Hyma Synthesis Pvt. Ltd. HPLC-grade acetonitrile (MeCN), tetrahydrofuran (THF), Chloroform ($CHCl_3$), dimethylformamide (DMF), TFE (Trifluoroethanol) and methanol (MeOH) were used and dried with 4Å molecular sieves. Without any further purification, all other compounds that were acquired were utilized just as they were received.

2. Experimental procedure and compound characterisation

2.1. Synthesis and characterisation of 2-iodobenzamides 21a-j

Iodobenzamide was made in compliance with the documented protocol.[45]

To a mixture of 2-iodobenzoic acid **19** (2.5 g, 10.0 mmol, 1.0 equiv) and thionyl chloride (1.5 mL, 20.2 mmol, 2.0 equiv) in CH_2Cl_2 (50 mL) was reacted with a catalytic amount of DMF (0.5 mL). The mixture was continuously stirred at room temperature for almost 1h. Afterward, the solvent was concentrated under reduced pressure at absolute temperature and another 50 mL of CH_2Cl_2 was added. Aniline derivatives **20** (11.1 mmol, 1.1 equiv) and triethylamine (TEA) (3.4 mL, 24.4 mmol, 2.4 equiv) were then introduced. Furtherly, the reaction mixture was continuously stirred at room temperature for 6-7h. Upon completion, the reaction was quenched with ice-cold water and the mixture was extracted with CH_2Cl_2 (3 × 20 mL). The overall organic layers were sequentially washed with 10% aqueous HCl and water. The corresponding organic phase was dried over sodium sulfate and the solvent was dried under reduced pressure at absolute temperature. With the use of silica gel, In column chromatography the crude product was refined using EtOAc/hexane (1:49) as the eluent, yielding products **21** with 64-94% efficiency.

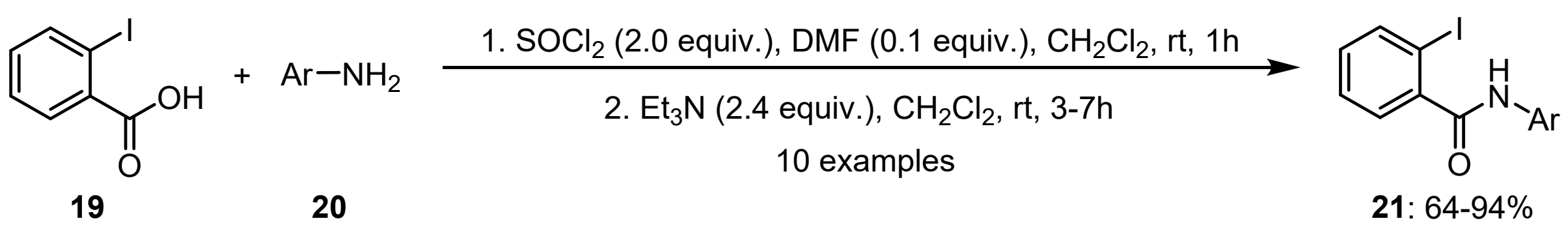

N-(Phenyl)-2-iodobenzamide (21a)[39,46]

Light brown solid, yield: 2.742 g, 8.47 mmol (85%), mp: 138-140 °C (Lit.[39] mp 136-139 °C), R_f = 0.8 (EtOAc-hexane 1:49); IR (KBr): ν = 3276 (NH) cm^{-1}, 1712 (C=O); GC-MS (EI): m/z = 323 [M^{+}.]; ^{1}H NMR

(400 MHz, CDCl3): δ = 7.83 (brs, 1H, NH), 7.74 (d, *J* = 8.0Hz, 1H, ArH), 7.52 (d, *J* = 7.6 Hz, 2H, ArH), 7.31 (dd, J_1 = 1.2 Hz, J_2 = 6.4 Hz, 1H, ArH), 7.24 (t, *J* = 7.6 Hz, 3H, ArH), 7.06 (t, *J* = 7.2 Hz, ArH), 6.98 (dt, J_1 = 1.2 Hz, J_2 = 8.0 Hz, 1H, ArH); ^{13}C NMR (100 MHz, CDCl3): δ = 167.4, 142.0, 139.9, 137.7, 131.4, 129.0 (2C), 128.5, 128.3, 124.8, 120.2 (2C), 92.5;

_N_-(2-fluorophenyl)-2-iodobenzamide (21b)[47]

White solid, yield: 2.181 g, 6.40 mmol (64%), mp: 160-162 °C (Lit. mp 161-163 °C), R_f = 0.5 (EtOAc-hexane 1:49); IR (KBr): *v* = 3242 (NH) cm-1, 1707 (C=O); GC-MS (EI): m/z = 341 [$M^{+\bullet}$]; ^{1}H NMR (400 MHz, CDCl3): δ = 8.39 (t, *J* = 7.6 Hz, 1H, ArH), 7.85 (d, *J* = 7.6 Hz, 1H, ArH), 7.64 (brs, 1H, NH), 7.44-7.46 (m, 1H, ArH), 7.36 (t, *J* = 7.6 Hz, 1H, ArH), 7.02-7.14 (m, 4H, ArH); ^{13}C NMR (100MHz, $CDCl_3$): δ = 167.1, 153.9, 151.5, 141.7, 140.2, 131.7, 128.5, 125.8, 125.0, 124.7, 121.9, 114.9, 92.4;

_N_-(4-fluorophenyl)-2-iodobenzamide (21c)[48]

White solid, yield: 2.557 g, 7.50 mmol (75%), mp: 150-152 °C (Lit. mp 151-152 °C), R_f = 0.6 (EtOAc-hexane 1:49); IR (KBr): *v* = 3122 (NH) cm^{-1}, 1683 (C=O); GC-MS (EI): m/z = 341 [$M^{+\bullet}$]; ^{1}H NMR (400 MHz, CDCl3): δ = 7.92 (brs, 1H, NH), 7.70-7.80 (m, 1H, ArH), 7.37-7.46 (m, 2H, ArH), 7.08-7.31 (m, 2H, ArH), 6.87-7.05 (m, 3H, ArH); ^{13}C NMR (100MHz, $CDCl_3$): δ = 167.5, 158.5, 141.8, 133.6 (2C), 131.5, 128.4 (2C), 122.2, 122.1, 115.6, 92.5;

_N_-(3-chlorophenyl)-2-iodobenzamide (21d)[49]

Pale yellow solid, yield: 2.744 g, 7.72 mmol (77%), mp: 170-172 °C (Lit. mp 169-170 °C), R_f = 0.3 (EtOAc-hexane 1:49); IR (KBr): *v* = 3322 (NH) cm^{-1}, 1721 (C=O); GC-MS (EI): m/z = 357 [$M^{+\bullet}$], 359 [$M^{+\bullet}$ + 2]; ^{1}H NMR (400 MHz, $CDCl_3$): δ = 7.97 (dd, J_1 = 0.8 Hz, J_2 = 7.90 (dd, J_1 = 1.6 Hz, J_2 = 6.4 Hz, 1H, ArH), 7.79 (d, *J* = 7.6 Hz, 1H, ArH), 7.67 (brs, 1H, NH), 7.36-7.39 (m, 1H, ArH), 7.18-7.21 (m, 2H, ArH), 7.11(dt, J_1 = 1.6 Hz, J_2 = 7.6 Hz, 1H, ArH), 7.03-7.07 (m, 1H, ArH), 7.2 Hz, 1H, ArH); ^{13}C NMR (100MHz, $CDCl_3$): δ = 167.4, 141.9, 140.1, 138.6, 134.8, 131.7, 130.1, 128.6, 128.4, 128.0, 124.9, 120.2, 118.1;

_N_-(4-chlorophenyl)-2-iodobenzamide (21e)[39]

Pale yellow solid, yield: 2.856 g, 8.01 mmol (80%), mp: 140-142 °C (Lit. mp 138-141 °C), R_f = 0.4 (EtOAc-hexane 1:49); IR (KBr): *v* = 3289 (NH) cm^{-1}, 1744 (C=O); GC-MS (EI): m/z = 357 [$M^{+\bullet}$], 359 [$M^{+\bullet}$ + 2]; ^{1}H NMR (400 MHz, $CDCl_3$): δ = 7.81-7.84 (m, 1H, ArH), 7.49-7.52 (m, 3H, NH, ArH), 7.41-7.43 (m, 1H, ArH), 7.32-7.37 (m, 1H, ArH), 7.21-7.28 (m, 2H, ArH), 7.05-7.09 (m, 1H, ArH); ^{13}C NMR (100MHz, $CDCl_3$): δ = 167.3, 141.8, 140.1, 136.1, 131.7, 129.2(2C), 128.6, 128.4, 121.4(2C), 92.3;

_N_-(4-bromophenyl)-2-iodobenzamide (21f)[50]

Yellow solid, yield: 3.82 g, 8.32 mmol (83%), mp: 148-150 °C (Lit. mp 150-153 °C), R_f = 0.6 (EtOAc-hexane 1:49); IR (KBr): *v* = 3250 (NH) cm^{-1}, 1705 (C=O); GC-MS (EI): m/z = 401 [$M^{+\bullet}$], 403 [$M^{+\bullet}$ + 2]; ^{1}H NMR (400 MHz, $CDCl_3$): δ = 7.96 (dd, J_1 = 0.8 Hz, J_2 = 7.2 Hz, 1H, ArH), 7.90 (dd, J_1 = 1.6 Hz, J_2 = 6.6 Hz, 1H, ArH), 7.73 (dt, *J* = 8.0 Hz, 1H, ArH), 7.72 (brs, 1H, ArH), 7.25-7.50 (m, 3H, ArH), 7.11 (dt, J_1 = 1.2 Hz, J_2 = 6.4 Hz, 1H, ArH), 7.03 (dt, J_1 = 0,8 Hz, J_2 = 8.0 Hz, 1H, ArH); ^{13}C NMR (100MHz, $CDCl_3$): δ = 167.5, 141.7, 140.0, 136.6, 133.4, 132.0 (2C), 131.7, 128.5, 128.0 (2C), 121.8, 92.3;

_N_-(4-cyanophenyl)-2-iodobenzamide (21g)[51]

White solid, yield: 2.74 g, 7.94 mmol (79%), mp: 153-155 °C (Lit. mp 154-155 °C), R_f = 0.4 (EtOAc-hexane 1:49); IR (KBr): *v* = 3123 (NH) cm^{-1}, 2209 (CN) cm^{-1}, 1697 (C=O); GC-MS (EI): m/z = 348 [$M^{+\bullet}$];

^{1}H NMR (400 MHz, $CDCl_3$): δ = 8.00 (dd, J_1 = 0.8 Hz, J_2 = 7.2 Hz, 2H, ArH), 7.95 (dd, J_1 = 1.6 Hz, J_2 = 8.0 Hz, 2H, ArH), 7.36-7.42 (m, 2H, ArH), 7.13 (tt, J_1 = 1.2 Hz, J_2 = 8.0 Hz, 2H, ArH), 5.23 (brs, 1H, NH); ^{13}C NMR (100MHz, $CDCl_3$): δ = 171.1, 141.9 (3C), 133.6 (2C), 133.2, 132.1 (2C), 128.1 (2C), 121.7, 94.7, 53.5;

_N_-(4-methylphenyl)-2-iodobenzamide (21h)[39,50]

White solid, yield: 2.89 g, 8.61 mmol (86%), mp: 139-141 °C (Lit. mp 137-140 °C), R_f = 0.4 (EtOAc-hexane 1:49); IR (KBr): *v* = 3223 (NH) cm^{-1}, 1731 (C=O); GC-MS (EI): m/z = 337 [M^+.]; ^{1}H NMR (400 MHz, $CDCl_3$): δ = 7.94-7.99 (m, 3H, ArH,), 7.46 (brs, 1H, NH), 7.36-7.46 (m, 2H, ArH), 7.18-7.19 (m, 1H, ArH), 7.07-7.14 (m, 2H, ArH), 2.38 (s, 3H, Me); ^{13}C NMR (100MHz, $CDCl_3$): δ = 170.9, 141.9, 133.5, 131.5, 129.7, 128.1, 127.5, 123.2, 120.2 (2C), 94.7, 30.1;

_N_-(4-methoxyphenyl)-2-iodobenzamide (21i)[39,50]

White solid, yield: 3.31 g, 9.40 mmol (94%), mp: 156-158^{0}C (Lit. mp 158-161^{0}C), R_f = 0.3 (EtOAc-hexane 1:49); IR (KBr): *v* = 3307 (NH) cm^{-1}, 1689 (C=O); GC-MS (EI): m/z = 353 [M^+**.**]; ^{1}H NMR (400 MHz, $CDCl_3$): δ = 7.81 (d, *J* = 8.0 Hz, 1H, ArH), 7.44-7.47 (m, 3H, NH, ArH), 7.39-7.42 (m, 1H, ArH), 7.32 (dt, J_1 = 0.4 Hz, J_2 = 7.6 Hz, 1H, ArH), 7.05 (dd, J_1 = 1.2 Hz, J_2 = 8.0 Hz, 1H, ArH), 6.81 (d, *J* = 8.0 Hz, 2H, ArH), 3.74 (s, 3H, OMe); ^{13}C NMR (100MHz, $CDCl_3$): δ = 167.2, 156.9, 142.2, 139.9, 131.4 (2C), 130.6, 128.5, 128.3, 122.0 (2C), 114.3, 92.5, 55.6;

_N_-(4-butylphenyl)-2-iodobenzamide (21j)

White solid, yield: 3.29 g, 8.70 mmol (87%), mp: 168-170 °C (Lit. mp 158-161 C), R_f = 0.3 (EtOAc-hexane 1:49); IR (KBr): *v* = 3290 (NH) cm^{-1}, 1711 (C=O); GC-MS (EI): m/z = 379 [M^+.]; ^{1}H NMR (400 MHz, $CDCl_3$): δ = 7.93 (brs, 1H, NH), 7.68 (d, *J* = 8.0 Hz, 1H, ArH), 7.39 (d, *J* = 8.0 Hz, 2H, ArH), 7.24 (d, *J* = 7.2 Hz, 1H, ArH), 7.17 (t, *J* = 7.6 Hz, 1H, ArH), 7.02 (d, *J* = 7.6 Hz, 2H, ArH), 6.92 (t, *J* = 7.6, 1H, ArH), 2.49 (t, *J* = 7.6 Hz, 2H, CH_2), 1.40-1.55 (m, 2H, CH_2), 1.19-1.30 (m, 2H, CH_2), 0.8 (t, *J* = 7.2 Hz, 3H, CH_3); ^{13}C NMR (100MHz, $CDCl_3$): δ = 167.4, 142.0, 139.9, 139.5, 135.4, 131.3 (2C), 128.9, 128.4, 128.2, 120.4 (2C), 92.7, 35.2, 133.7, 22.4, 4.1;

2.2. Synthesis and characterisation of 2-(1-arylethynyl) benzamides 17a-k

2-(1-arylethynyl) benzamides was made in compliance with the documented protocol.[41]

To a mixture of N-phenyl-2-iodobenzamide **21a** (1.0 mmol) and DMF (6 mL) was prepared and $Pd(PPh_3)Cl_2$ (0.025 g, 3.5 mol%), CuI (0.030 g, 16 mol%) and Et_3N (0.55 mL, 4.0 mmol, 2.0 equiv.) were added under a nitrogen atmosphere. After stirring the reaction mixture at room temperature for almost 10 minutes, phenylacetylene **22** (0.22 mL, 2.0 mmol, 2.0 equiv.) was then introduced. The reaction mixture was then heated at 85°C and maintained for nearly 3-5 hours. while the reaction progress was being monitored by TLC. After the completion of the reaction, ice-cold-water (10 mL) was added to the reaction mixture and it was neutralized with a saturated NH_4Cl solution (5 mL). The mixture was extracted with EtOAc (3 x 30 mL) and the overall organic extracts were washed with water (3 x 30 mL). The corresponding organic phase was dried over sodium sulfate, filtered and concentrated under vacuum. In column chromatography the crude product was refined using silica gel using an EtOAc/hexane (2:48) mixture as the eluent. The obtained products were identified as N-phenyl-2-(1-arylethynyl) benzamides **17a** through spectroscopic analysis.

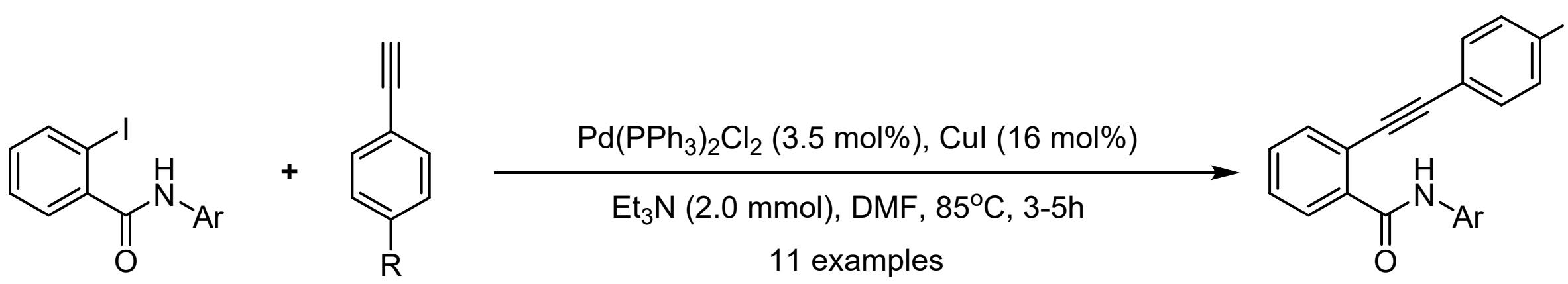

N-Phenyl-2-(phenylethynyl) benzamide (17a)[39,52]

White solid, yield: 0.213 g, 0.723 mmol (72%), mp: 148-150 °C (Lit. mp 147-150 °C), R_f = 0.6 (EtOAc-hexane 2:48); IR (KBr): *v* = 3224 (NH) cm^{-1}, 1726 (C=O); GC-MS (EI): m/z = 297 [M^+.]; ^1H NMR (400 MHz, $CDCl_3$): δ = 9.12 (brs, 1H, NH), 8.02-8.07 (m, 1H, ArH), 7.53- 7.61 (m, 3H, ArH), 7.41-7.50 (m, 4H, ArH), 7.21-7.35 (m, 5H, ArH), 7.05 (t, *J* = 7.2 Hz, 1H, ArH); ^{13}C NMR (100MHz, $CDCl_3$): δ = 164.4, 137.9, 135.9, 133.5, 131.7, (2C), 130.9, 130.3, 129.3, 129.1 (3C), 128.7 (2C), 124.5, 121.9, 120.0 (2C), 119.6, 96.6, 87.3;

N-(2-Fluorophenyl)-2-(phenylethynyl) benzamide (17b)

White solid, yield: 0.214 g, 0.681 mmol (68%), mp: 170-172°C, R_f = 0.3 (EtOAc-hexane 2:48); IR (KBr): *v* = 3256 (NH) cm^{-1}, 1706 (C=O); GC-MS (EI): m/z = 315 [M^+.]; ^1H NMR (400 MHz, $CDCl_3$): δ = 9.18 (brs, 1H, NH), 7.88 (d, *J* = 7.6 Hz, 1H, ArH), 7.61 (tt, J_1 = 0.8 Hz, J_2 = 7.6 Hz, 1H, ArH), 7.48 (tt, J_1 = 0.4 Hz, J_2 = 7.6 Hz, 1H, ArH), 6.95-7.02 (m, 2H, ArH), 6.81-6.91 (m, 4H, ArH), 6.76-6.79 (m, 2H, ArH), 6.71-6.75 (m, 1H, ArH); ^{13}C NMR (100MHz, $CDCl_3$): δ = 167.5, 134.2, 133.1, 132.6, 129.7, 129.3, 129.2, 128.8 (2C), 127.7, 127.2 (2C), 126.7, 123.9, 123.8, 123.7, 119.6, 116.0, 115.8, 129.4, 107.7;

N-(4-Fluorophenyl)-2-(phenylethynyl) benzamide (17c)

White solid, yield: 0.220 g, 0.701 mmol (70%), mp: 141-143 °C, R_f = 0.3 (EtOAc-hexane 2:48); IR (KBr): *v* = 3074 (NH) cm^{-1}, 1711 (C=O); GC-MS (EI): m/z = 315 [M^+.]); ^1H NMR (400 MHz, $CDCl_3$): δ = 9.13 (brs, 1H, NH), 8.02-8.04 (m, 1H, ArH), 7.51-7.61 (m, 3H, ArH), 7.38-7.44 (m, 4H, ArH), 7.29-7.33 (m, 3H, ArH), 6.91-6.99 (m, 2H, ArH); ^{13}C NMR (100MHz, $CDCl_3$): δ = 164.4, 133.5, 131.6, 131.0, 130.3, 129.4, 129.1 (2C), 128.7, 127.4 (2C), 121.7, 121.6 (2C), 119.6, 115.6 (2C), 115.2, 107.6, 96.6, 87.2;

N-(3-Chlorophenyl)-2-(phenylethynyl) benzamide (17d)[39]

White solid, yield: 0.248 g, 0.752 mmol (75%), mp: 119-121 °C (Lit. mp 119-120 °C), R_f = 0.2 (EtOAc-hexane 2:48); IR (KBr): *v* = 3322 (NH) cm^{-1}, 1704 (C=O); GC-MS (EI): m/z = 331 [M^+.], 333 [M^+. + 2]; ^1H NMR (400 MHz, $CDCl_3$): δ = 9.20 (brs, 1H, NH), 7.85 (d, *J* = 7.6 Hz, 1H, ArH), 7.77 (d, *J* = 7.6 Hz, 1H, ArH), 7.60 (t, *J* = 7.6 Hz, 1H, ArH), 7.38-7.41 (m, 2H, ArH), 7.27-7.34 (m, 2H, ArH), 6.88-6.94 (m, 4H, ArH), 6.78-6.81 (m, 2H, ArH); ^{13}C NMR (100MHz, $CDCl_3$): δ = 164.5, 136.8, 134.1, 133.7, 133.4, 132.7, 131.6, 129.4, 129.0 (2C), 128.9, 128.7, 127.6 (2C), 127.4, 127.0, 126.7, 125.4, 124.0, 119.4, 107.9;

N-(4-Chlorophenyl)-2-(phenylethynyl) benzamide (17e)[39]

White solid, yield: 0.254 g, 0.770 mmol (77%), mp: 160-162 °C (Lit. mp 161-163 °C), R_f = 0.4 (EtOAc-hexane 2:48); IR (KBr): *v* = 3239 (NH) cm^{-1}, 1731 (C=O); GC-MS (EI): m/z = 331 [M^+.], 333 [M^+. + 2]; ^1H NMR (400 MHz, $CDCl_3$): δ = 9.18 (brs, 1H, NH), 7.84 (d, *J* = 7.6 Hz, 1H, ArH), 7.76 (d, *J* = 7.4 Hz, 1H, ArH), 7.59 (t, *J* = 7.6 Hz, 1H, ArH), 7.46 (t, *J* = 7.4 Hz, 1H, ArH), 7.39-7.42 (m, 1H, ArH), 7.26-7.36 (m, 2H, ArH),

7.17-7.20 (m, 1H, ArH), 6.86-6.97 (m, 2H, ArH), 6.76-6.78 (m, 2H, ArH); ^{13}C NMR (100MHz, $CDCl_3$): δ = 167.8, 138.5, 132.7, 131.6 (2C), 131.1, 129.4, 129.2, 128.7, 128.4 (2C), 128.3 (2C), 127.4 (2C), 126.9, 124.8, 123.9, 121.2, 119.5, 107.8;

_N_-(4-Bromophenyl)-2-(phenylethynyl) benzamide (17f)

Pale yellow solid, yield: 0.300 g, 0.801 mmol (80%), mp: 150-152 °C, R_f = 0.5 (EtOAc-hexane 2:48); IR (KBr): *v* = 3270 (NH) cm^{-1}, 1751 (C=O); GC-MS (EI): m/z = 375 [M^{+}**.**], 377 [M^{+}**.** + 2]; ^{1}H NMR (400 MHz, $CDCl_3$): δ = 7.86 (d, *J* = 7.6 Hz, 1H, ArH), 7.80 (d, *J* = 7.4 Hz, 1H, ArH), 7.61 (t, *J* = 7.6 Hz, 1H, ArH), 7.57 (t, *J* = 7.4 Hz, 1H, ArH), 7.51-7.56 (m, 2H, NH, ArH), 7.45-7.49 (m, 2H, ArH), 7.21-7.26 (m, 1H, ArH), 6.90-6.97 (m, 3H, NH, ArH), 6.72-6.74 (m, 2H, ArH); ^{13}C NMR (100MHz, $CDCl_3$): δ = 167.8, 138.5, 132.7, 131.6, 131.1, 128.7 (2C), 128.4, 128.3 (2C), 127.4, 126.9, 123.9 (2C), 122.9, 121.2, 119.5, 107.8;

_N_-(4-Cyanophenyl)-2-(phenylethynyl) benzamide (17g)

White solid, yield: 0.254 g, 0.790 mmol (79%), mp: 144-146 °C, R_f = 0.3 (EtOAc-hexane 2:48); IR (KBr): *v* = 2268 (C≡N) cm^{-1}, 1723 (C=O); GC-MS (EI): m/z = 322 (M^{+}**.**]; ^{1}H NMR (400 MHz, $CDCl_3$): δ = 7.85 (d, *J* = 7.6 Hz, 1H, ArH), 7.76-7.81 (m, 3H, NH, ArH), 7.68 (t, *J* = 8.0 Hz, 1H, ArH), 7.64 (t, *J* = 7.2 Hz, 2H, ArH), 7.44-7.48 (m, 1H, ArH), 7.39-7.43 (m, 1h, ArH), 7.31-7.38 (m, 3H, ArH), 7.24 (t, *J* = 7.6 Hz, 2H, ArH); ^{13}C NMR (100MHz, $CDCl_3$): δ = 167.1, 144.6, 140.6, 134.9, 134.5, 133.1, 130.2 (2C), 129.8, 128.9, 128.8 (2C), 128.4, 128.2, 126.0, 125.6, 125.2, 123.4, 120.5, 119.8, 107.1, 101.8;

_N_-(4-methylphenyl)-2-(phenylethynyl) benzamide (17h)[39,53]

White solid, yield: 0.252 g, 0.810 mmol (81%), mp: 165-167 °C, (Lit. mp 166-169 °C), R_f = 0.6 (EtOAc-hexane 2:48); IR (KBr): *v* = 3290 (NH) cm^{-1}, 1737 (C=O); GC-MS (EI): m/z = 311 [M^{+}**.**]; ^{1}H NMR (400 MHz, $CDCl_3$): δ = 7.84-7.86 (m, 1H, ArH), 7.75-7.80 (m, 2H, NH, ArH), 7.61-7.69 (m, 2H, ArH), 7.43-7.47 (m, 4H, ArH), 7.31-7.38 (m, 3H, ArH), 7.13 (t, *J* = 8.4 Hz, 2H, ArH), 2.33 (s, 3H, CH_3); ^{13}C NMR (100MHz, $CDCl_3$): δ = 167.1, 144.6, 140.6, 134.9 (2C), 134.5 (2C), 133.1, 130.2, 129.8, 128.9, 128.8, 128.5, 125.6 (2C), 125.3, 123.4, 119.8, 107.1, 101.8, 25.9;

_N_-(4-methoxyphenyl)-2-(phenylethynyl) benzamide (17i)[39,53]

White solid, yield: 0.277 g, 0.851 mmol (85%), mp: 153-155 °C, (Lit. mp 155-156 °C), R_f = 0.3 (EtOAc-hexane 2:48); IR (KBr): *v* = 3272 (NH) cm^{-1}, 1650 (C=O); GC-MS (EI): m/z = 327 [M^{+}**.**]; ^{1}H NMR (400 MHz, $CDCl_3$): δ = 9.04 (s, 1H, NH), 8.01-8.03 (m, 1H, ArH), 7.53-7.59 (m, 1H, ArH), 7.46-7.52 (m, 2H, ArH), 7.35-7.41 (m, 4H, ArH), 7.25-7.30 (m, 3H, ArH), 6.71-6.80 (m, 2H, ArH), 3.71 (s, 3H, OMe); ^{13}C NMR (100MHz, $CDCl_3$): δ = 164.3, 156.5, 136.0, 133.5, 131.7 (2C), 131.2, 130.8, 130.2, 129.3, 129.1, 128.7 (2C), 127.2, 121.7 (2C), 119.5, 114.2 (2C), 96.4, 87.4, 55.5;

_N_-(4-Butylphenyl)-2-(phenylethynyl) benzamide (17j)

White solid, yield: 0.289 g, 0.820 mmol (82%), mp: 178-180 °C, R_f = 0.4 (EtOAc-hexane 2:48); IR (KBr): *v* = 3243 (NH) cm^{-1}, 1683 (C=O); GC-MS (EI): m/z = 353 [M^{+}**.**]; ^{1}H NMR (400 MHz, $CDCl_3$): δ = 9.08 (s, 1H, NH), 7.53-7.56 (m, 1H, ArH), 7.43-7.48 (m, 2H, ArH), 7.36-7.41 (m, 4H, ArH), 7.21-7.29 (m, 4H, ArH), 7.05 (d, *J* = 8.0 Hz, 2H, ArH), 2.50 (t, *J* = 7.6 Hz, 2H, CH_2), 1.46-1.54 (m, 2H, CH_2), 1.21-1.31 (m, 2H, CH_2), 0.84 (t, *J* = 7.2 Hz, 3H, CH_3); ^{13}C NMR (100MHz, $CDCl_3$): δ = 163.2, 138.1, 134.9, 134.5, 132.4, 130.7, 129.7, 129.3 (2C), 128.2, 128.0 (2C), 127.9 (2C), 127.6, 120.9 (2C), 119.0, 118.5, 95.4, 86.3, 34.1, 32.6, 21.3, 12.9;

2.3. Synthesis and characterisation of 6*H*-isoindolo[2,1-a] indol-6-ones 18a-k through ultrasonication and ball milling

6*H*-isoindolo[2,1-a] indol-6-ones 18a-k: General procedure (a)

A stirred solution of **17a** (500 mg, 1.683 mmol, 1.0 equiv.) in trifluoroethanol (1.0 mL) was treated with phenyliodine diacetate (PIDA) (704.9 mg, 2.188 mmol, 1.3 equiv.) in an oven-dried round-bottom flask at room temperature. The reaction mixture was then subjected to ultrasonic irradiation in a sonication bath (33 kHz, 100 W) for 15-30 min at room temperature, while the progress of the reaction was monitored by TLC analysis. The starting material exhibited under short-wavelength UV light (254 nm), whereas the product showed fluorescence under long-wavelength UV light (365 nm). Upon completion of the reaction, the solvent was removed under reduced pressure and water (5.0 mL) was added to the residue. The resulting mixture was extracted with ethyl acetate (3 × 10 mL) and the combined organic layers were dried over anhydrous Na_2SO_4, filtered and concentrated under reduced pressure. The crude product was subsequently purified by column chromatography using ethyl acetate/hexane (1:49) as the eluent to afford the corresponding 11-aryl-6*H*-isoindolo[2,1-a] indol-6-ones, which were characterized by detailed spectroscopic analysis.

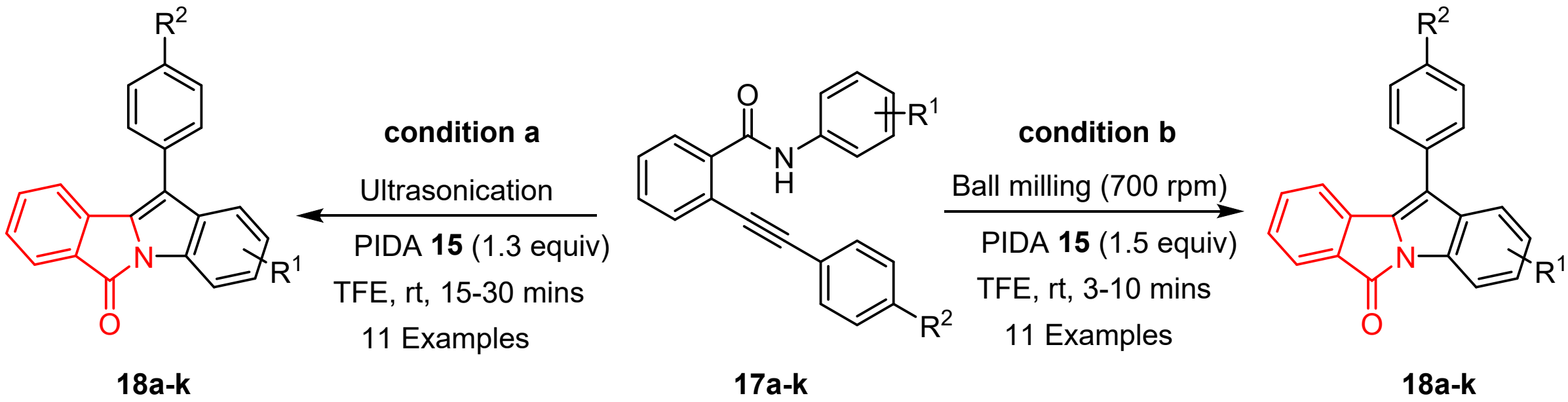

6*H*-isoindolo[2,1-a] indol-6-ones 18a-k: General procedure (b)

A stirred solution of **17a** (500 mg, 1.683 mmol, 1.0 equiv.) in trifluoroethanol (1.0 mL) was treated with phenyliodine diacetate (PIDA) (813.38 mg, 2.525 mmol, 1.5 equiv.) in an oven-dried round-bottom flask at room temperature. The reaction mixture was then subjected to FRITSCH Pulverisette planetary ball milling with jar size of 10ml and agate balls (13 balls) with a rotation speed of 700rpm for 3-10 min at room temperature, while the progress of the reaction was monitored by TLC analysis. The starting material exhibited under short-wavelength UV light (254 nm), whereas the product showed fluorescence under long-wavelength UV light (365 nm). Upon completion of the reaction, the solvent was removed under reduced pressure and water (5.0 mL) was added to the residue. The resulting mixture was extracted with ethyl acetate (3 × 10 mL) and the combined organic layers were dried over anhydrous Na_2SO_4, filtered and concentrated under reduced pressure. The crude product was subsequently purified by column chromatography using ethyl acetate/hexane (1:49) as the eluent to afford the corresponding 11-aryl-6*H*-isoindolo[2,1-a]indol-6-ones, which were characterized by detailed spectroscopic analysis.

11-phenyl-6*H*-isoindolo[2,1-a] indol-6-one (18a)[39]

Yellow solid, yield: 0.123 g, 0.420 mmol (89%), mp: 220-222 °C (Lit. mp 221^{0}C), R_f = 0.6 (EtOAc-hexane 1:49); IR (KBr): *v* = 1721 (C=O) cm^{-1}; GC-MS (EI): *m/z* = 295 [M^+.]; **HRMS (ESI): m/z calculated for $C_{21}H_{14}NO$ $[M+H]^+$ = 296.1070, found $[M+H]^+$ = 296.1064; error = -2.0 ppm;** ^{1}H NMR (400 MHz, $CDCl_3$): δ = 7.87 (d, *J* = 8.0 Hz, 1H, ArH), 7.69 (d, *J* = 7.2 Hz, 1H, ArH), 7.61-7.63 (m, 2H, ArH), 7.45-7.50 (m, 4H, ArH), 7.32 (t, *J* = 7.6 Hz, 1H, ArH), 7.20-7.27 (m, 2H, ArH), 7.09 (t, *J* = 6.8 Hz, 1H, ArH); ^{13}C NMR (100 MHz, $CDCl_3$): δ = 162.6, 134.7, 134.2, 134.0, 133.9, 133.8, 133.6, 132.2, 129.0 (2C), 128.9 (2C), 128.8, 128.3, 126.8, 125.4, 124.1, 121.3, 121.2, 120.6, 113.5;

4-Fluoro-11-phenyl-6*H*-isoindolo[2,1-a] indol-6-one (18b)

Pale yellow solid, yield: 0.103 g, 0.330 mmol (66%), mp: 191-196 °C, R_f = 0.4 (EtOAc-hexane 1:49); IR (KBr): *v* = 1746 (C=O) cm^{-1}; GC-MS (EI): m/z = 313 [M^+.]; ^{1}H NMR (400 MHz, $CDCl_3$): δ = 7.87-7.89 (m, 2H, ArH), 7.71-7.73 (m, 2H, ArH), 7.34-7.39 (m, 3H, ArH), 7.29 (t, *J* = 7.2 Hz, 1H, ArH), 7.16-7.22 (m, 4H, ArH); ^{13}C NMR (100 MHz, $CDCl_3$): δ = 166.6, 159.1, 156.7, 134.5 (2C), 131.9, 130.9, 130.7, 129.9, 126.9, 125.3, 124.9, 124.7, 123.9 (2C), 121.5, 119.4, 119.3, 117.9, 116.9, 116.6;

2-fluoro-11-phenyl-6*H*-isoindolo[2,1-a] indol-6-one (18c)

Yellow solid, yield: 0.122 g, 0.390 mmol (82%), mp: 181-184 °C, R_f = 0.5 (EtOAc-hexane 1:49); IR (KBr): v = 1716 (C=O) cm-1; GC-MS (EI): m/z = 313 [M^+.]**; HRMS (ESI): m/z calculated for $C_{21}H_{13}FNO$ $[M+H]^+$ = 314.0976, found $[M+H]^+$ = 314.0987; error = +3.6 ppm;** ^{1}H NMR (400 MHz, CDCl3): δ = 7.80-7.83 (m, 1H, ArH), 7.71 (d, *J* = 7.2 Hz, 1H, ArH), 7.60 (d, *J* = 7.6 Hz, 2H, ArH), 7.49 (t, *J* = 7.6 Hz, 3H, ArH), 7.39-7.42 (m, 1H, ArH), 7.35 (t, *J* = 7.6 Hz, 1H, ArH), 7.26 (t, *J* = 7.6 Hz, 1H, ArH), 7.15 (dd, J_1 = 2.0 Hz, J_2 = 8.6 Hz, 1H, ArH), 6.98 (tt, J_1 = 2.0 Hz, J2 = 8.8 Hz, 1H, ArH); ^{13}C NMR (100 MHz, CDCl3): δ = 162.3, 159.0, 135.7, 135.1, 135.0, 134.5, 133.8, 133.7, 131.8, 130.1, 129.2, 129.1, 128.8, 128.6, 125.5, 114.3, 114.3, 114.2, 114.1, 113.8, 107.6, 107.4;

3-Chloro-11-phenyl-6*H*-isoindolo[2,1-a] indol-6-one (18d)

Yellow solid, yield: 0.115 g, 0.349 mmol (72%), mp: 180-184 °C, R_f = 0.3 (EtOAc-hexane 1:49); IR (KBr): v = 1736 (C=O) cm-1; GC-MS (EI): m/z = 329 [M^+.], 331 [M^+. + 2] ; ^{1}H NMR (400 MHz, CDCl3): δ = 7.84 (d, *J* = 8.0 Hz, 1H, ArH), 7.69 7.74 (m, 1H, ArH), 7.61 (d, *J* = 7.6 Hz, 1H, ArH), 7.48-7.53 (m, 2H, ArH), 7.35-7.45 (m, 2H, ArH), 7.23-7.29 (m, 2H, ArH), 7.19 (s, 1H, ArH), 7.09 (d, *J* = 8.0 Hz, 1H, ArH), 7.05 (d, *J* = 8.0 Hz, 1H, ArH), ^{13}C NMR (100 MHz, CDCl3): δ = 112.0, 121.3, 121.9 (2C), 124.5, 125.4, 125.5, 125.7, 126.1, 127.3, 127.9, 128.3, 128.5, 128.8, 129.1, 129.1, 129.2 (2C), 130.5, 133.9, 134.0, 167.8;

2-Chloro-11-phenyl-6*H*-isoindolo[2,1-a] indol-6-one (18e)[39]

Yellow solid, yield: 0.131 g, 0.401 mmol (84%), mp: 194-195 °C (Lit. mp 193-194 °C) R_f = 0.4 (EtOAc-hexane 1:49); IR (KBr): *v* = 1724 (C=O) cm^{-1}; GC-MS (EI): m/z = 329 [M^+.], 331 [M^+. + 2]; ^{1}H NMR (400 MHz, $CDCl_3$): δ = 7.78 (d, *J* = 8.8 Hz, 1H, ArH), 7.69 (d, *J* = 7.6 Hz, 1H, ArH), 7.59 (d, *J* = 7.2 Hz, 2H, ArH), 7.48 (t, *J* = 7.2 Hz, 3H, ArH), 7.38-7.45 (m, 2H, ArH), 7.35 (t, *J* = 7.2 Hz, 1H, ArH), 7.26(t, *J* = 7.6 Hz, 1H, ArH), 7.18-7.22(m, 1H, ArH); ^{13}C NMR (100 MHz, $CDCl_3$): δ = 162.3, 135.3, 135.2, 134.8, 133.8, 133.6, 131.9, 131.6, 129.7, 128.9 (2C), 128.6, 126.8, 125.6, 123.8, 121.0, 121.4, 119.7, 114.3;

2-Bromo-11-phenyl-6*H*-isoindolo[2,1-a] indol-6-one (18f)

Yellow solid, yield: 0.154 g, 0.415 mmol (85%), mp: 196-198 °C, R_f = 0.6 (EtOAc-hexane 1:49); IR (KBr): *v* = 1730 (C=O) cm^{-1}; GC-MS (EI): m/z = 373 [M^+.], 375 [M^+. + 2] ; ^{1}H NMR (400 MHz, $CDCl_3$): δ = 7.74 (d, *J* = 8.4 Hz, 1H, ArH), 7.71 (d, *J* = 7.6 Hz, 1H, ArH), 7.56-7.60 (m, 3H, ArH), 7.49 (t, *J* = 6.8 Hz, 3H, ArH),

7.39-7.43 (m, 1H, ArH), 7.34-7.38 (m, 2H, ArH), 7.27 (t, *J* = 7.2Hz, 1H, ArH); ^{13}C NMR (100 MHz, $CDCl_3$): δ = 162.4, 135.7, 135.1 (2C), 134.3, 133.9, 133.6, 132.3, 131.6, 129.5, 129.2, 128.9, 128.6, 125.9, 125.6, 124.0, 121.5 (2C), 119.6, 117.3, 114.7;

6-Oxo-11-phenyl-6*H*-isoindolo[2,1-a] indol-2-carbonitrile (18g)

Yellow solid, yield: 0.129 g, 0.405 mmol (87%), mp: 198-201 °C, R_f = 0.5 (EtOAc-hexane 1:49); IR (KBr): *v* = 2290 (C≡N) cm^{-1}, 1786 (C=O); GC-MS (EI): m/z = 320 [M^{+}**.**] ; ^{1}H NMR (400 MHz, $CDCl_3$): δ = 7.67-7.75 (m, 3H, ArH), 7.52-7.60 (m, 2H, ArH), 7.23-7.38 (m, 5H, ArH), 7.16 (t, *J* = 7.6 Hz, 1H, ArH), 6.25 (s, 1H, ArH); ^{13}C NMR (100 MHz, $CDCl_3$): δ = 167.1, 144.5, 140.5, 134.9, 134.5, 133.1, 130.1, 129.9, 129.8, 128.9, 128.8, 128.4, 128.2, 126.1 (2C), 125.5, 125.2, 123.3 (2C), 119.9, 107.1, 101.8;

2-methyl-11-phenyl-6*H*-isoindolo[2,1-a] indol-6-one (18h)[39]

Yellow solid, yield: 0.134 g, 0.435 mmol (91%), mp: 179-180 °C (Lit. mp 176-177 °C), R_f = 0.6 (EtOAc-hexane 1:49); IR (KBr): *v* = 1727 (C=O) cm^{-1}; GC-MS (EI): m/z 309 [M^{+}**.**] ; **HRMS (ESI): m/z calculated for $C_{22}H_{16}NO$ [M+H]$^{+}$ = 310.1226, found [M+H]$^{+}$ = 310.1233; error = +2.3 ppm;** ^{1}H NMR (400 MHz, $CDCl_3$): δ = 7.77 (d, *J* = 7.6 Hz, 1H, ArH), 7.74 (d, *J* = 7.2 Hz, 1H, ArH), 7.62-7.66 (m, 3H, ArH), 7.51-7.57 (m, 3H, ArH), 7.31-7.34 (m, 1H, ArH), 7.21-7.28 (m, 2H, ArH), 6.91 (t, *J* = 7.6 Hz, 1H, ArH), 2.83 (s, 3H, CH_3); ^{13}C NMR (100 MHz, $CDCl_3$): δ = 168.2, 141.2, 137.8, 136.3, 134.6, 134.1, 133.2, 130.4, 129.9, 129.7 (2C), 129.2, 128.6, 128.1, 127.5, 126.3 (2C), 126.1, 125.7, 115.3, 22.4;

2-methoxy-11-phenyl-6*H*-isoindolo[2,1-a] indol-6-one (18i)[39]

Yellow solid, yield: 0.147 g, 0.455 mmol (93%), mp: 192-194 °C (Lit. mp 195 °C), R_f = 0.2 (EtOAc-hexane 1:49); IR (KBr): *v* = 1747 (C=O) cm^{-1}; GC-MS (EI): m/z = 325 [M^{+}**.**]; ^{1}H NMR (400 MHz, $CDCl_3$): δ = 7.74 (d, *J* = 8.8 Hz, 1H, ArH), 7.65 (d, *J* = 7.6 Hz, 1H, ArH), 7.59 (d, *J* = 7.6 Hz, 2H, ArH), 7.43-7.49 (m, 2H, ArH), 7.38 (t, *J* = 7.6 Hz, 1H, ArH), 7.29 (t, *J* = 7.6 Hz, 1H, ArH), 7.25 (t, *J* = 7.6 Hz, 1H, ArH), 7.17-7.22 (m, 1H, ArH), 6.91-6.93 (m, 1H, ArH), 6.81-6.84 (m, 1H, ArH), 3.73 (s, 3H, OCH_3); ^{13}C NMR (100 MHz, $CDCl_3$): δ = 162.3, 157.0, 135.1, 133.9, 133.5, 132.2, 129.1, 128.9, 128.7 (2C), 128.4, 128.3, 125.3, 121.1 (2C), 120.3, 114.3, 114.0, 105.0, 55.8;

2-butyl-11-phenyl-6*H*-isoindolo[2,1-a] indol-6-one (18j)

Yellow solid, yield: 0.154 g, 0.440 mmol (89%), mp: 174-178 °C, R_f = 0.4 (EtOAc-hexane 1:49); IR (KBr): *v* = 1726 (C=O) cm^{-1}; GC-MS (EI): m/z = 351 [M^{+}**.**] ; ^{1}H NMR (400 MHz, $CDCl_3$): δ = 7.76 (d, *J* = 8.0 Hz, 1H, ArH), 7.66 (d, *J* = 7.6 Hz, 1H, ArH), 7.61 (t, *J* = 7.2 Hz, 2H, ArH), 7.45-7.49 (m, 3H, ArH), 7.38 (t, *J* = 7.6 Hz, 1H, ArH), 7.29 (t, *J* = 7.6 Hz, 1H, ArH), 7.16-7.23 (m, 2H, ArH), 7.06 (d, *J* = 8.0 Hz, 1H, ArH), 2.55 (d, *J* = 7.6 Hz, 2H, CH_2), 1.48-1.56 (m, 2H, CH_2), 1.23-1.32 (m, 2H, CH_2), 0.84 (t, *J* = 7.2 Hz, 3H, CH_3); ^{13}C NMR (100 MHz, $CDCl_3$): δ = 162.4, 139.0, 134.7, 134.4, 133.9, 133.5, 132.1, 129.0 (2C), 128.6, 128.3, 127.4, 125.3, 121.0, 120.7, 120.5, 113.2, 35.9, 34.2, 22.4, 14.0;

11-(4-butylphenyl)-2-methoxy-6*H*-isoindolo[2,1-a] indol-6-one (18k)

Yellow solid, yield: 0.169 g, 0.440 mmol (87%), mp: 184-187 °C, R_f = 0.4 (EtOAc-hexane 1:49); IR (KBr): *v* = 1726 (C=O) cm^{-1}; GC-MS (EI): m/z = 423 [M^{+}**.**] ; 1H NMR (400 MHz, $CDCl_3$): δ = 7.76 (d, J = 8.0 Hz, 1H, ArH), 7.67–7.72 (m, 2H, ArH), 7.52–7.58 (m, 1H, ArH), 7.43 (d, *J* = 8.0 Hz, 1H, ArH), 7.29–7.35 (m, 2H, ArH), 7.21–7.24 (m, 1H, ArH), 7.18 (d, *J* = 8.0 Hz, 1H, ArH), 6.95–6.98 (m, 2H, ArH), 6.82–6.85 (m, 1H, ArH), 3.74 (s, 3H, OCH_3), 2.63–2.66 (t, *J* = 7.6 Hz, 2H, CH_2), 1.60–1.65 (m, 2H, CH_2), 1.30–1.39 (m, 2H, CH_2), 0.89–0.91 (t, J = 7.2 Hz, 3H, CH_3); ^{13}C NMR (100 MHz, $CDCl_3$): δ = 162.2, 157.0, 143.2, 135.2, 128.6, 134.5, 133.8, 129.4, 129.0, 128.6, 125.9, 125.1, 122.1, 120.4, 115.8, 114.2, 106.2, 77.4, 77.0, 76.7, 55.8, 35.5, 33.6, 22.5, 14.0;

^{1}H NMR data of 11-phenyl-6*H*-isoindolo[2,1-a] indol-6-one (18a)

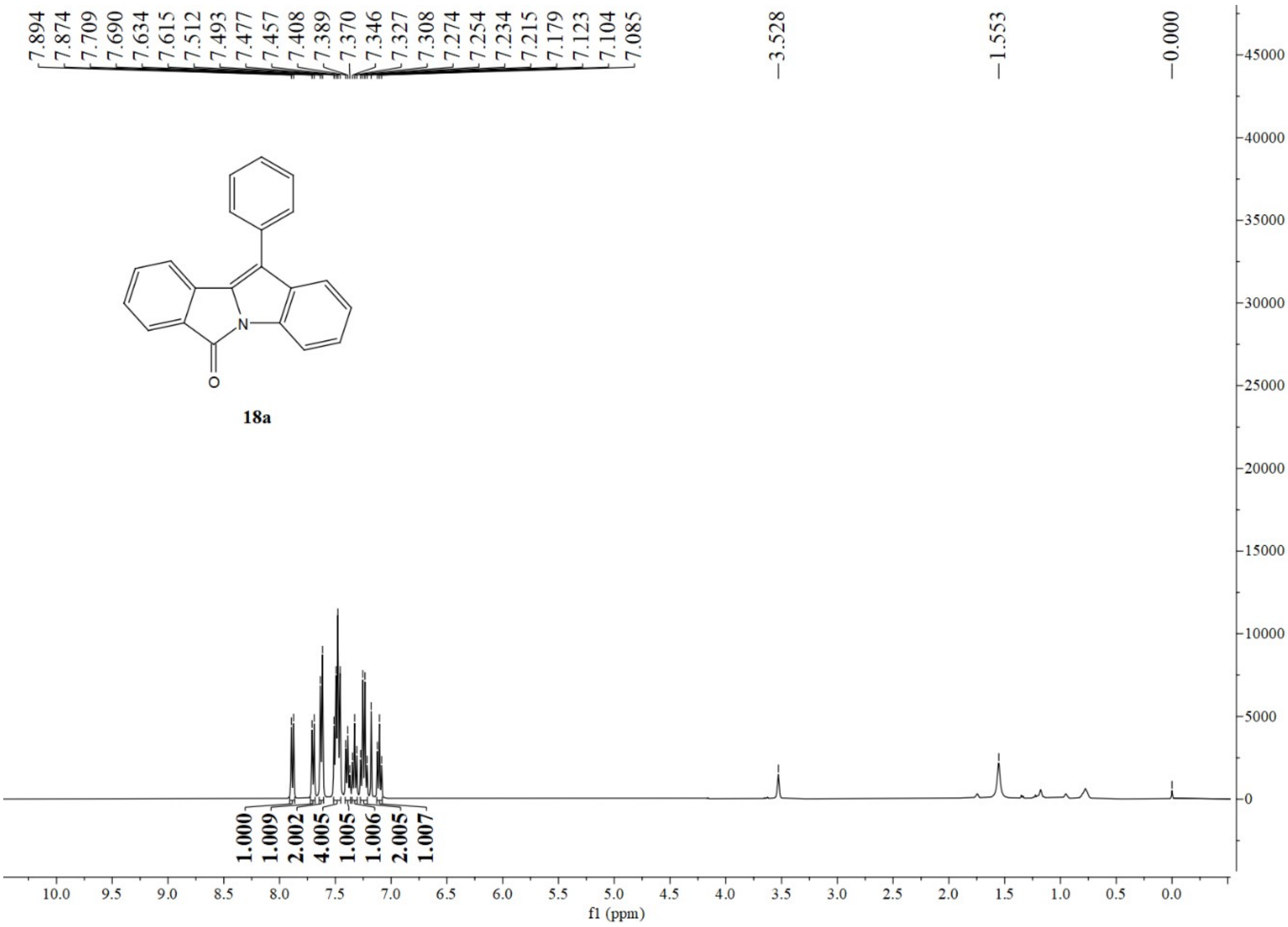

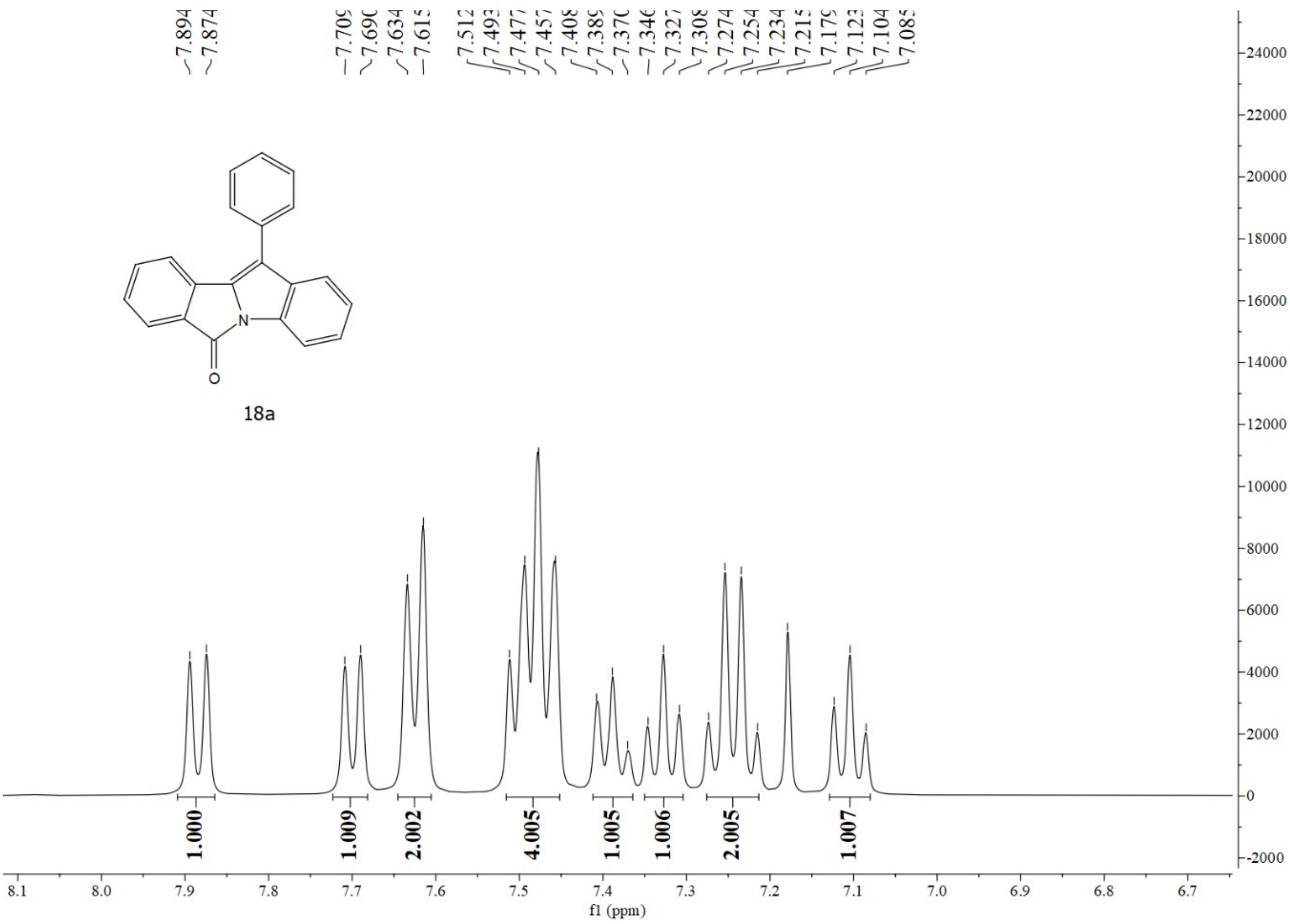

^{13}C NMR data of 11-phenyl-6*H*-isoindolo[2,1-a] indol-6-one (18a)

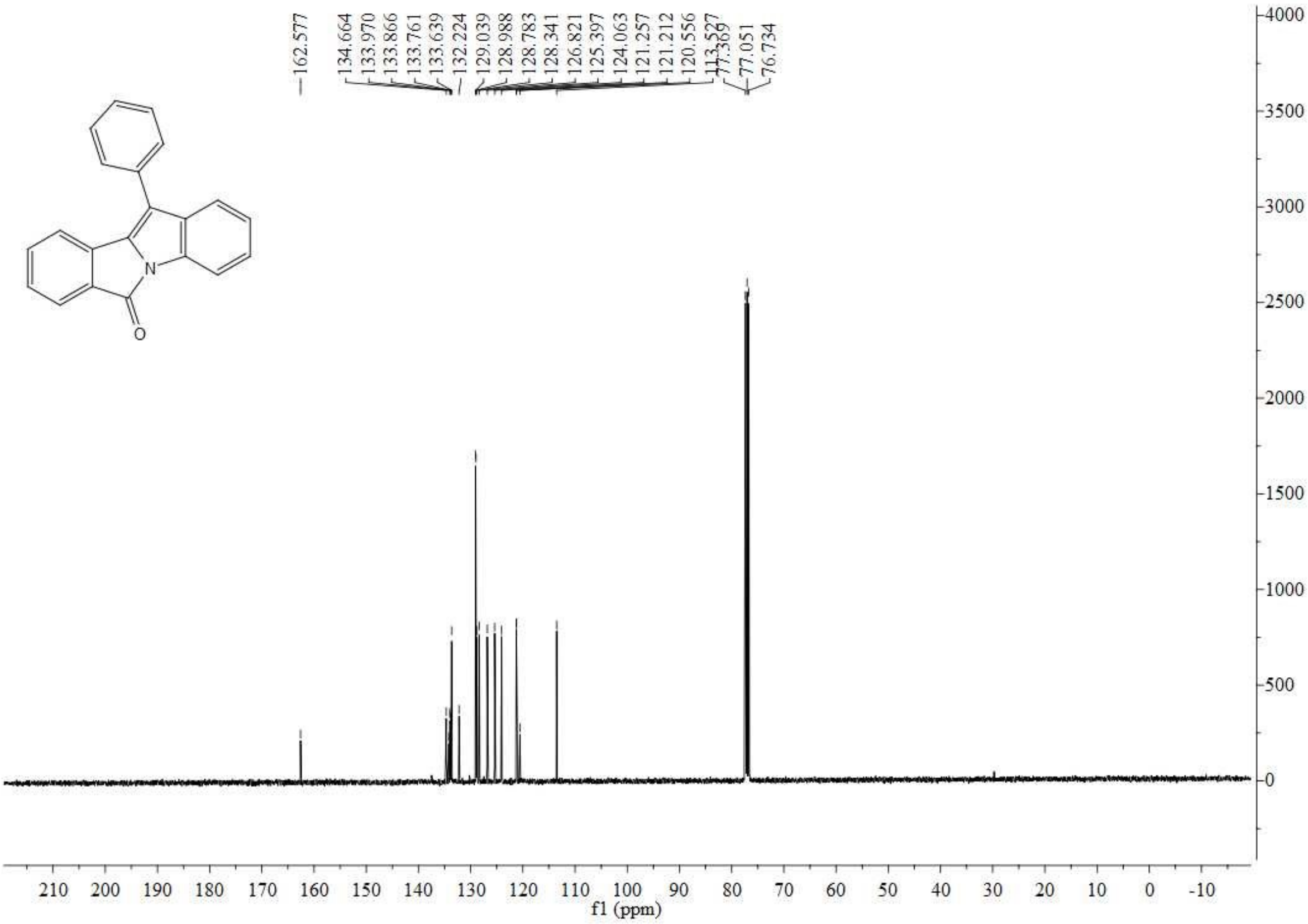

^{1}H NMR of 4-Fluoro-11-phenyl-6*H*-isoindolo[2,1-a] indol-6-one (18b)

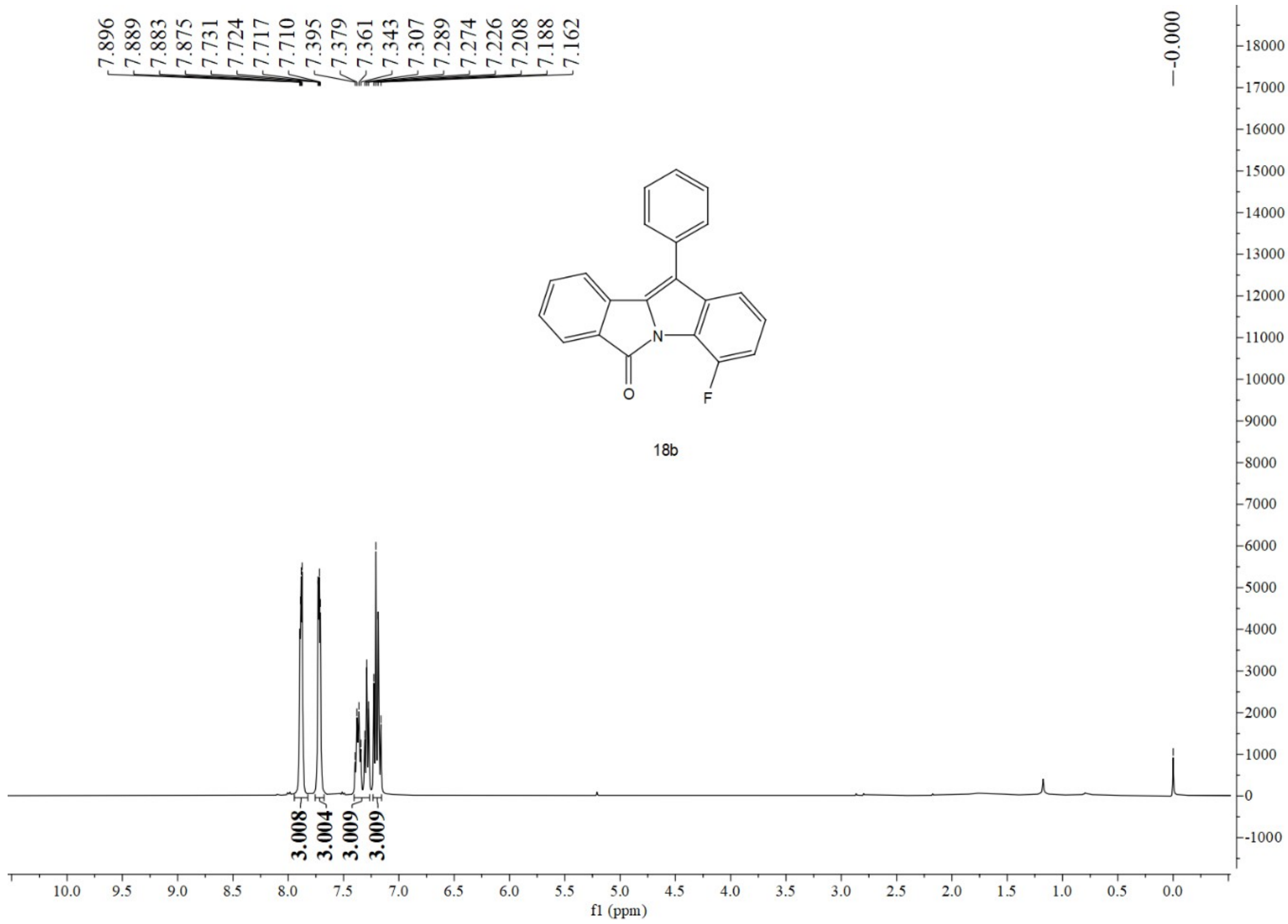

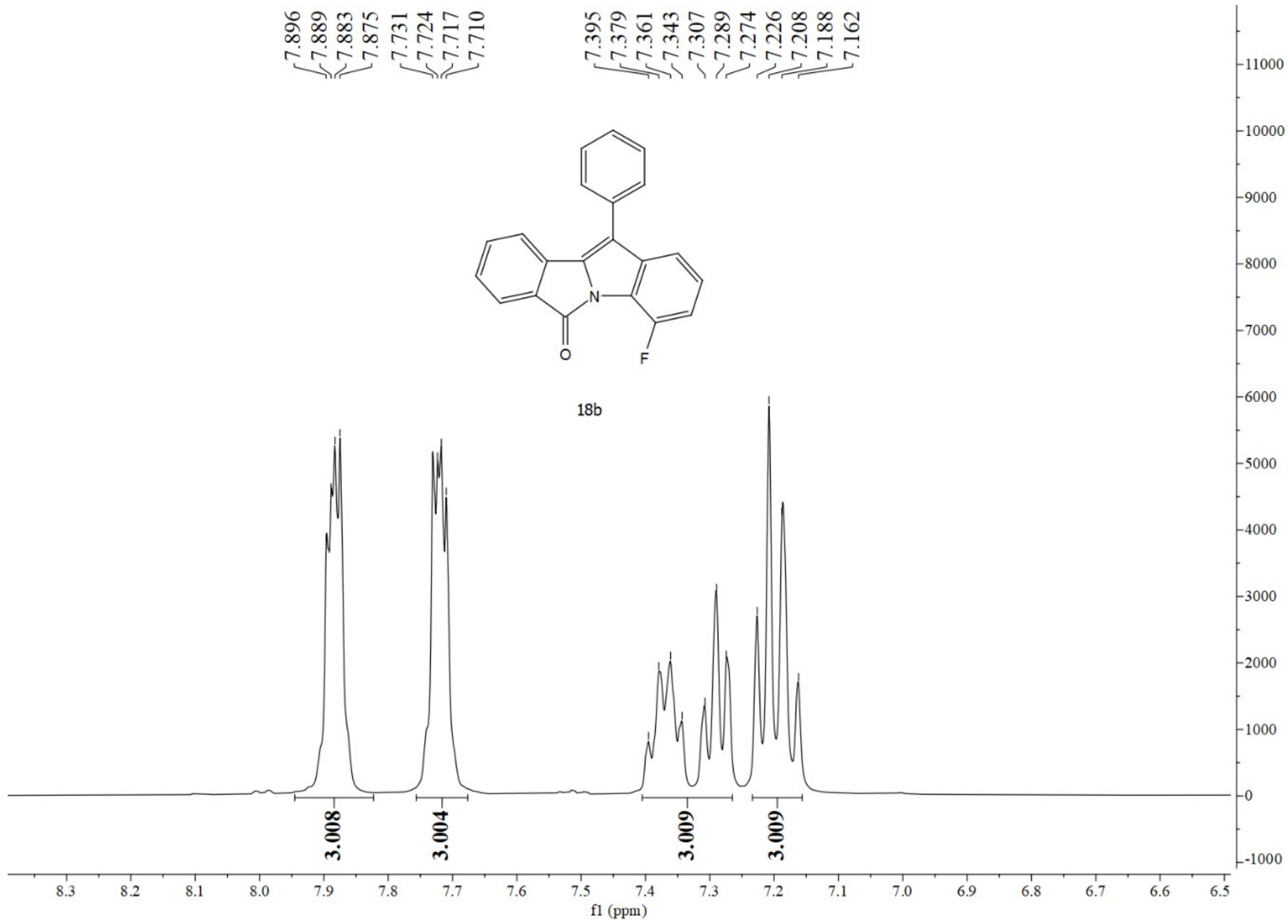

^{13}C NMR of 4-Fluoro-11-phenyl-6*H*-isoindolo[2,1-a] indol-6-one (18b)

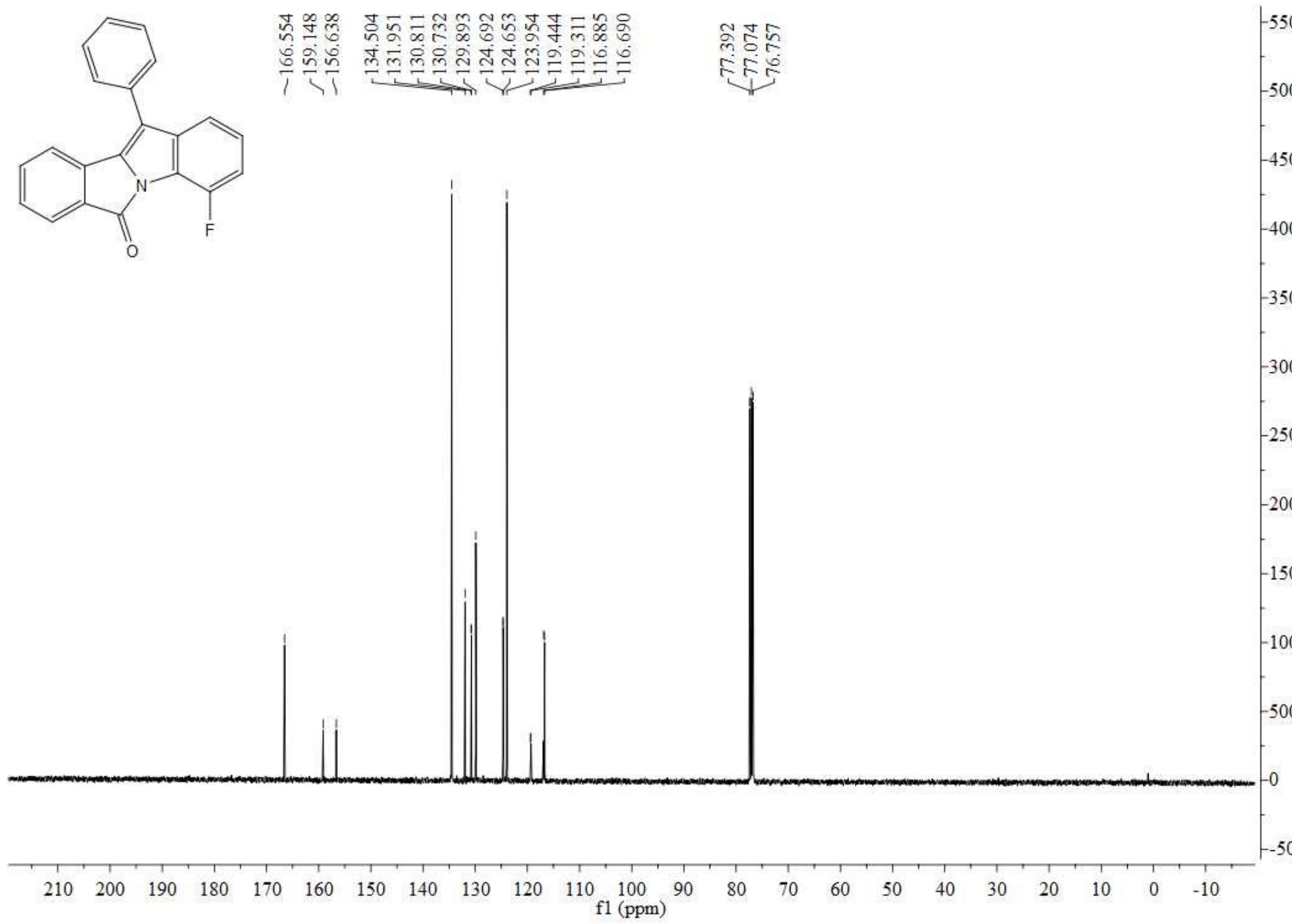

^{1}H NMR of 2-fluoro-11-phenyl-6H-isoindolo[2,1-a] indol-6-one (18c)

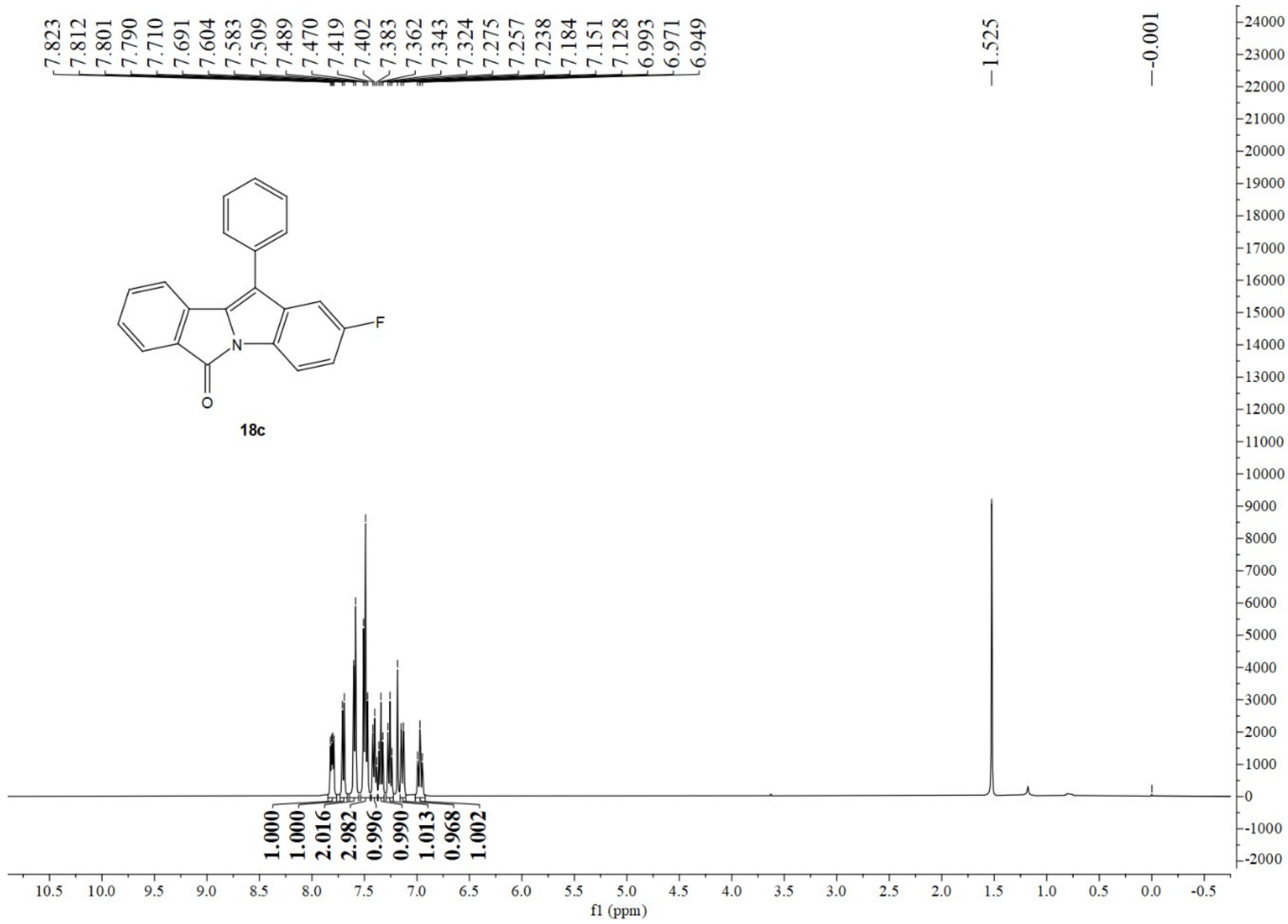

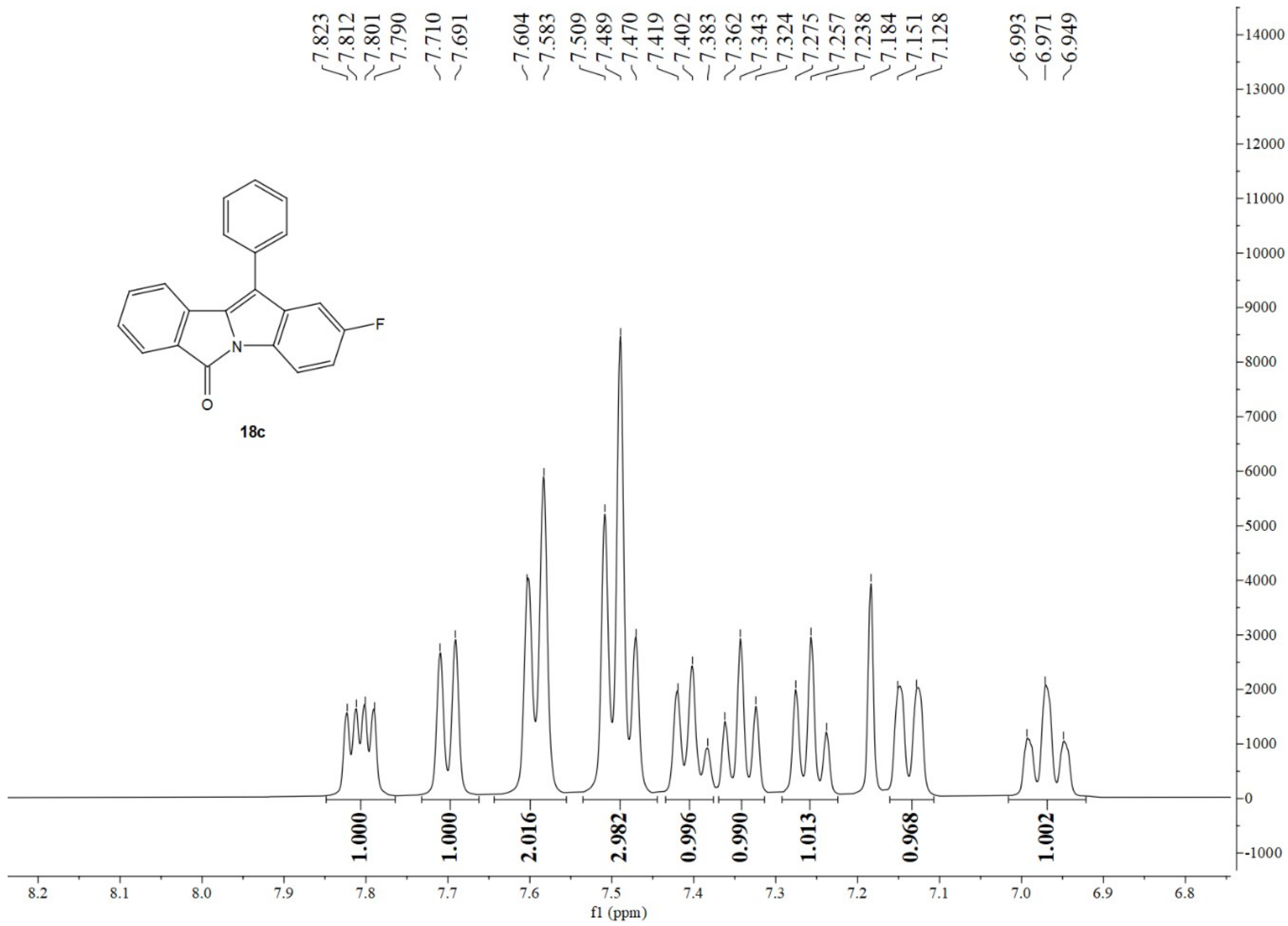

^{13}C NMR of 2-fluoro-11-phenyl-6H-isoindolo[2,1-a] indol-6-one (18c)

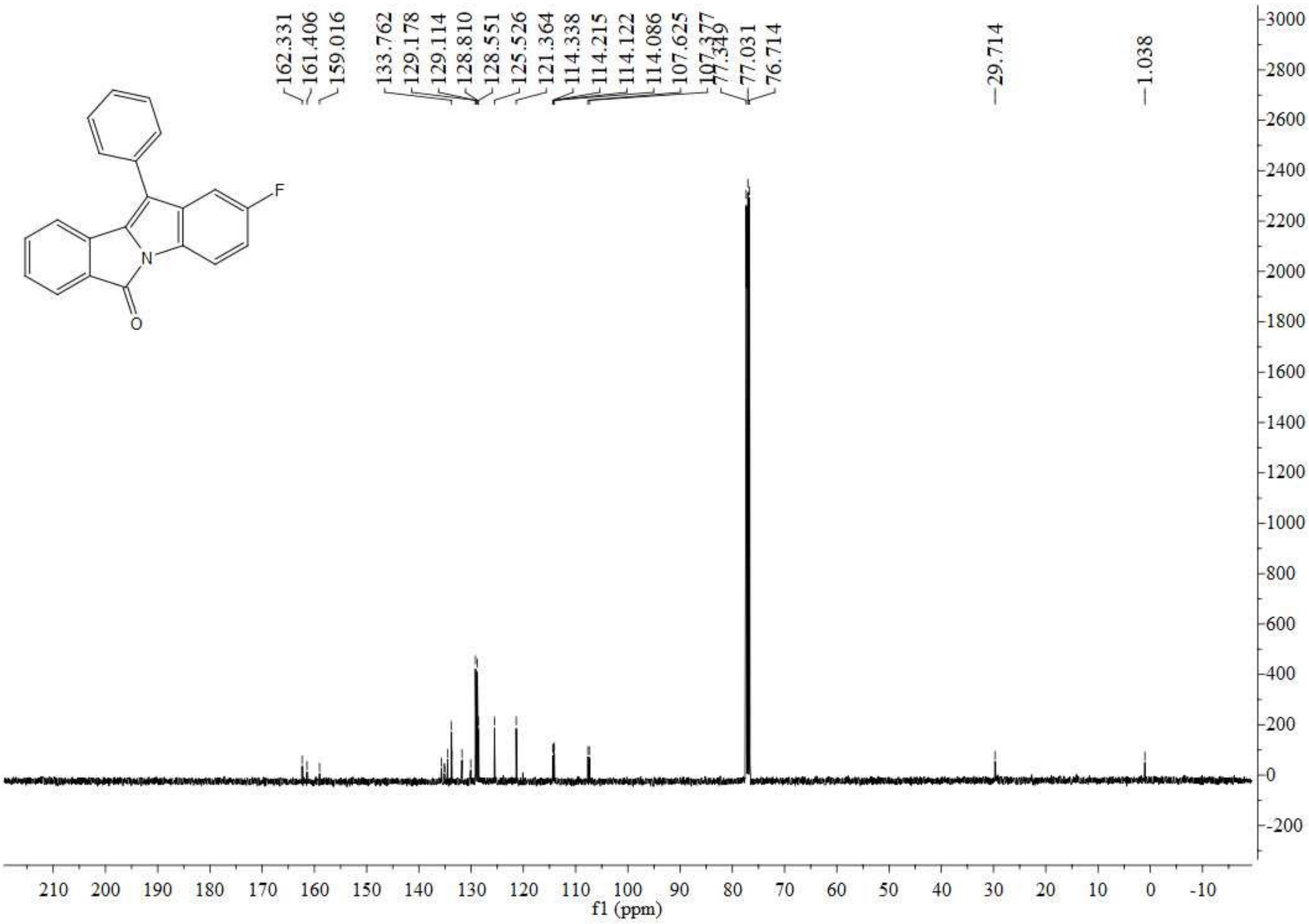

^{1}H NMR of 3-Chloro-11-phenyl-6H-isoindolo[2,1-a] indol-6-one (18d)

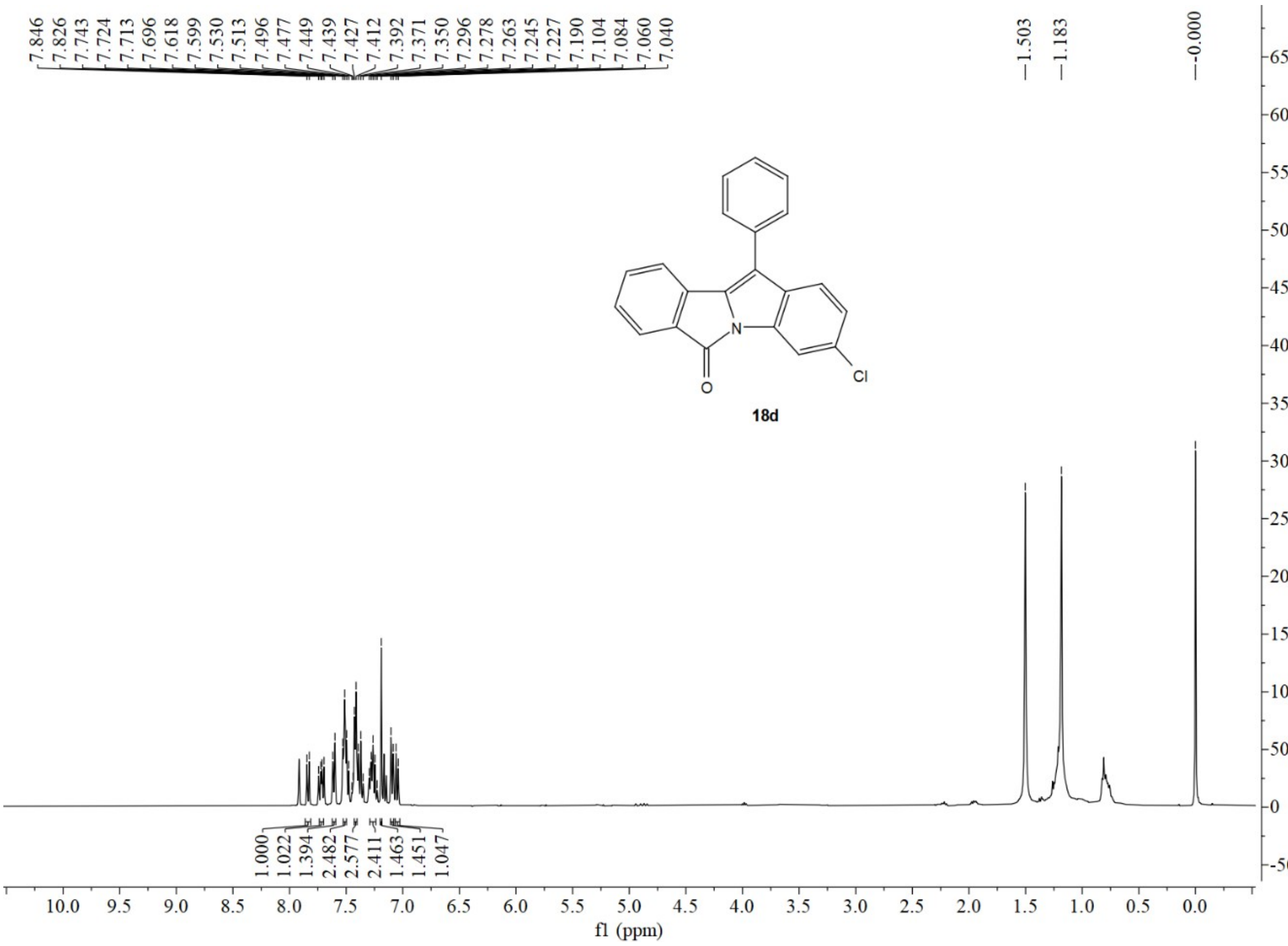

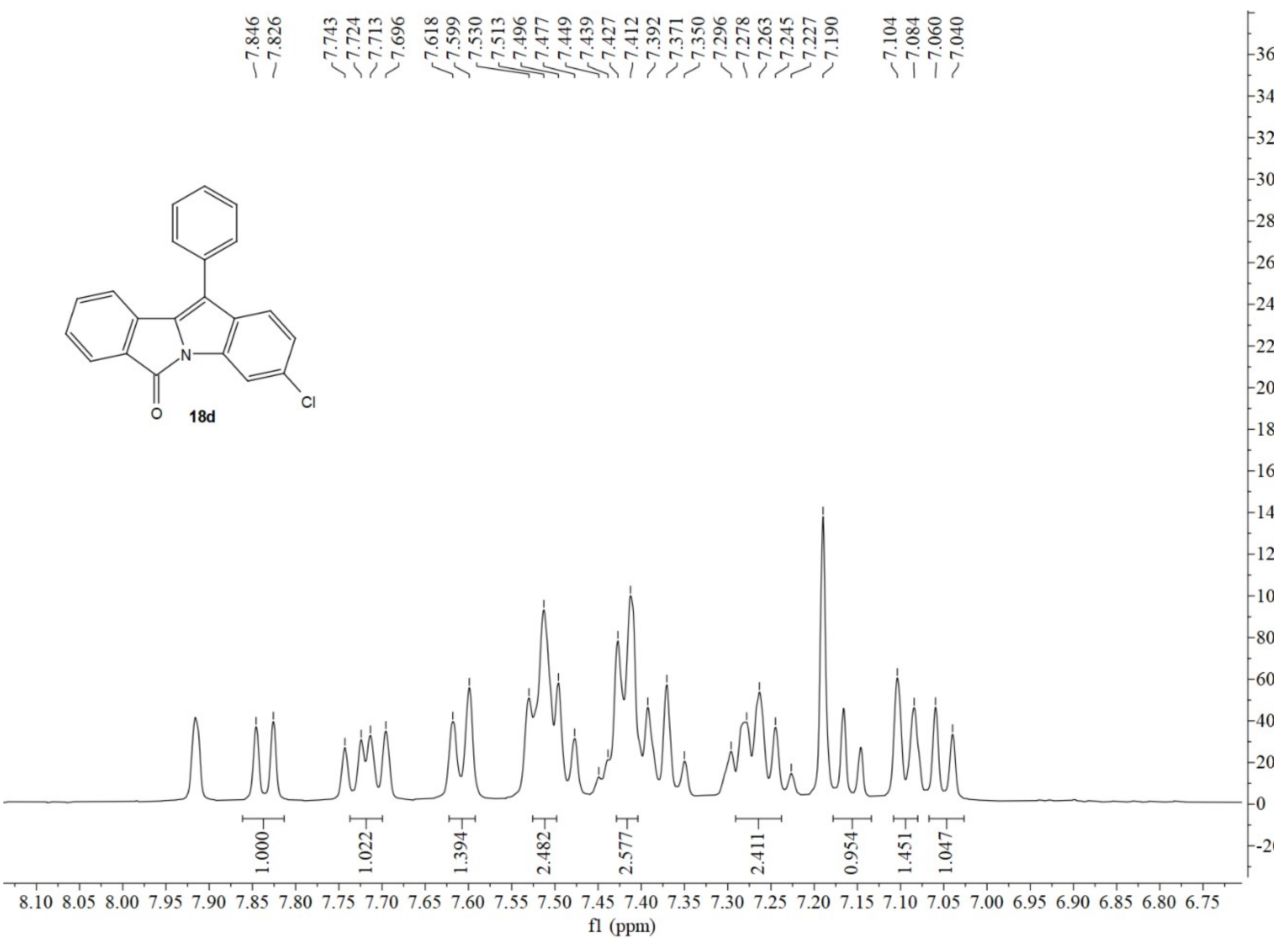

^{13}C NMR of 3-Chloro-11-phenyl-6H-isoindolo[2,1-a] indol-6-one (18d)

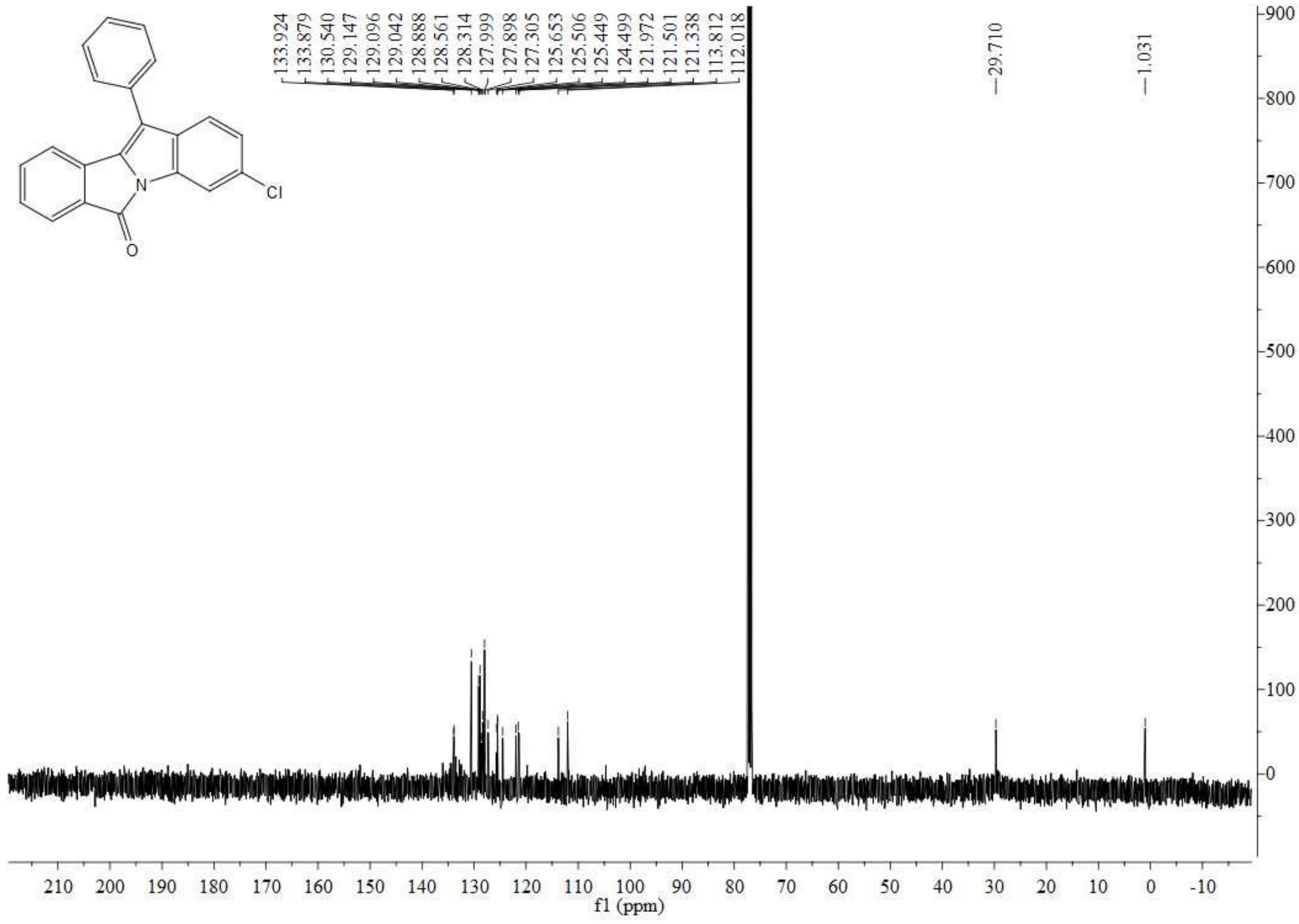

^{1}H NMR of 2-Chloro-11-phenyl-6H-isoindolo[2,1-a] indol-6-one (18e)

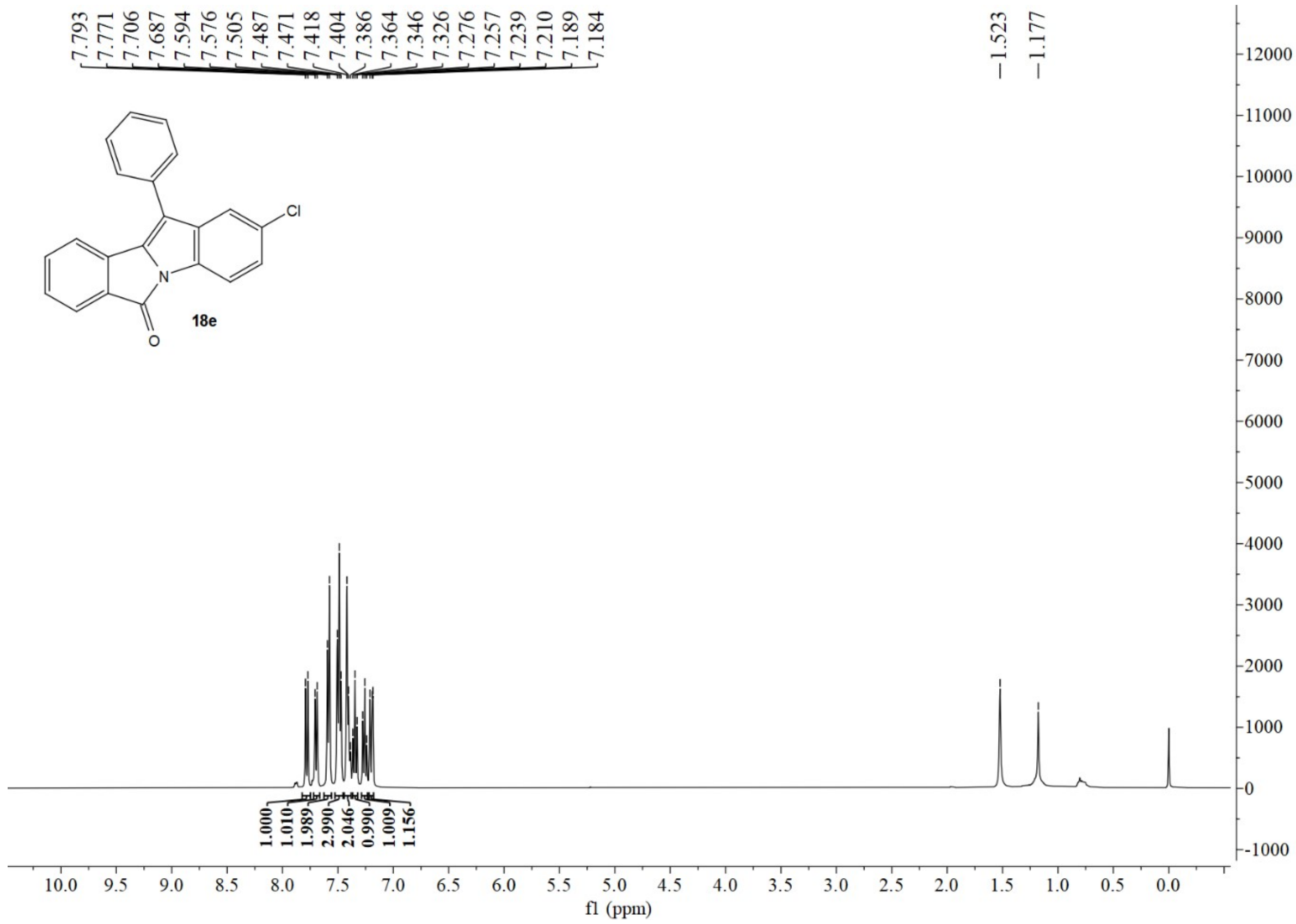

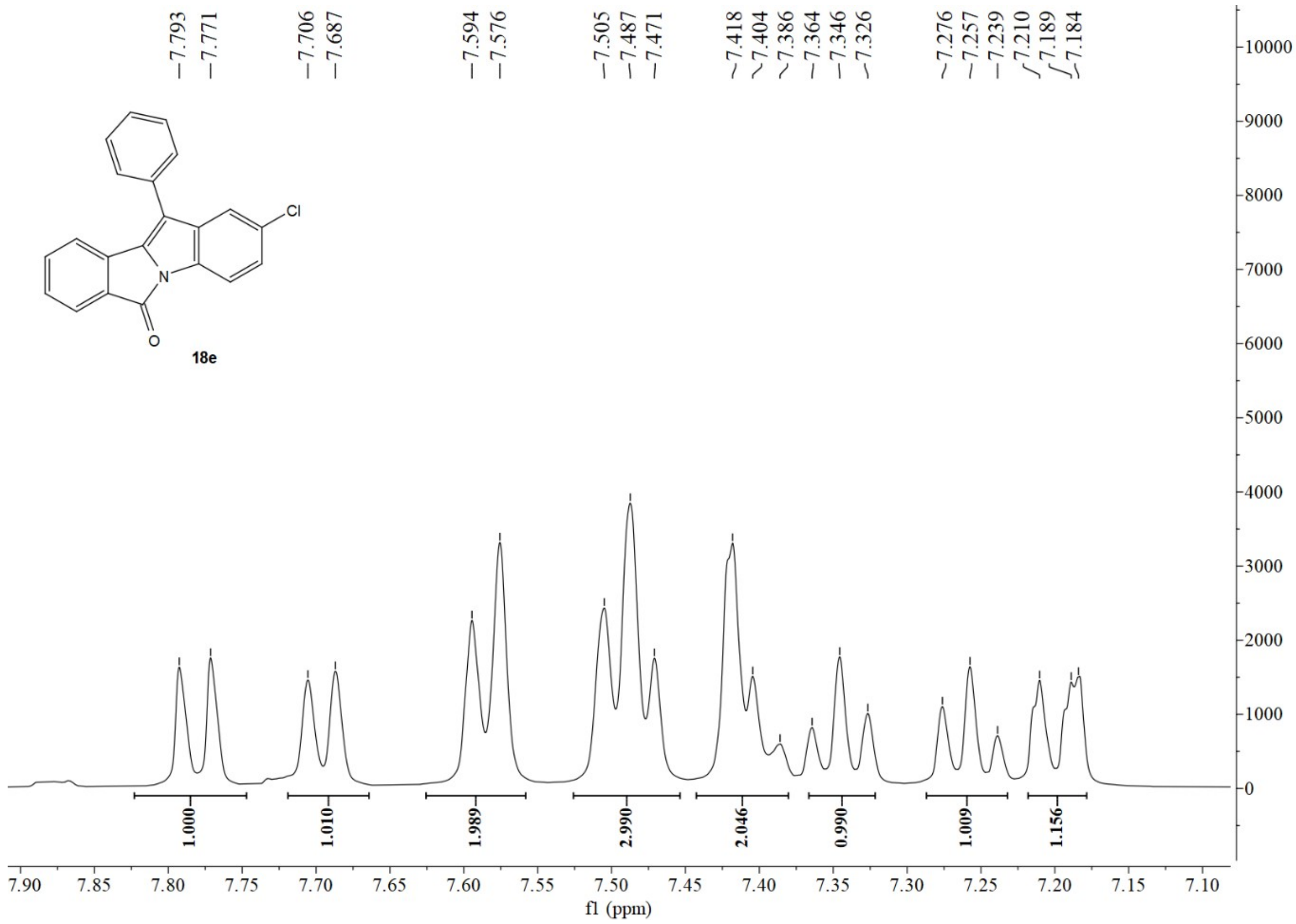

^{13}C NMR of 2-Chloro-11-phenyl-6H-isoindolo[2,1-a] indol-6-one (18e)

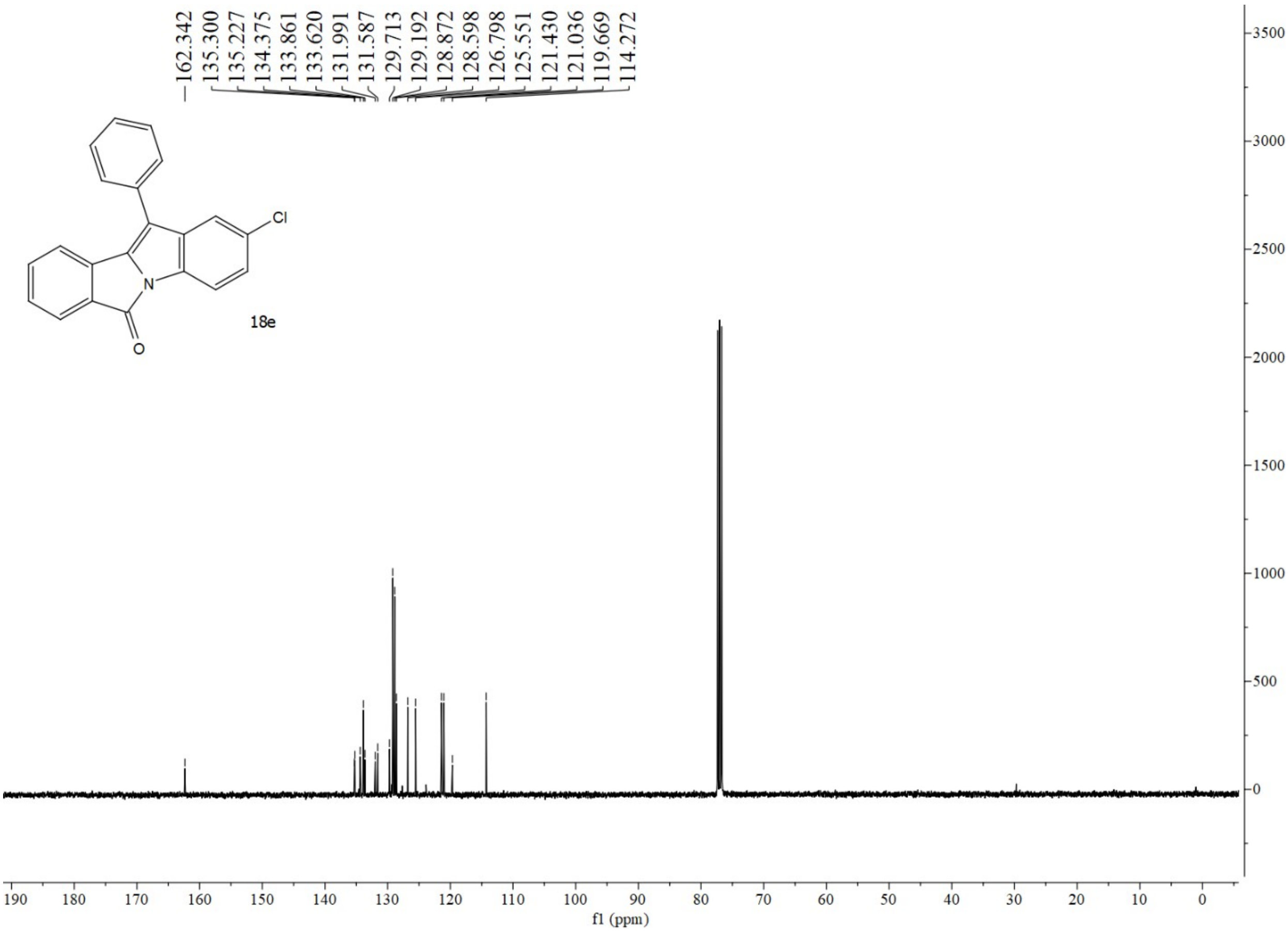

^{1}H NMR of 2-Bromo-11-phenyl-6H-isoindolo[2,1-a] indol-6-one (18f)

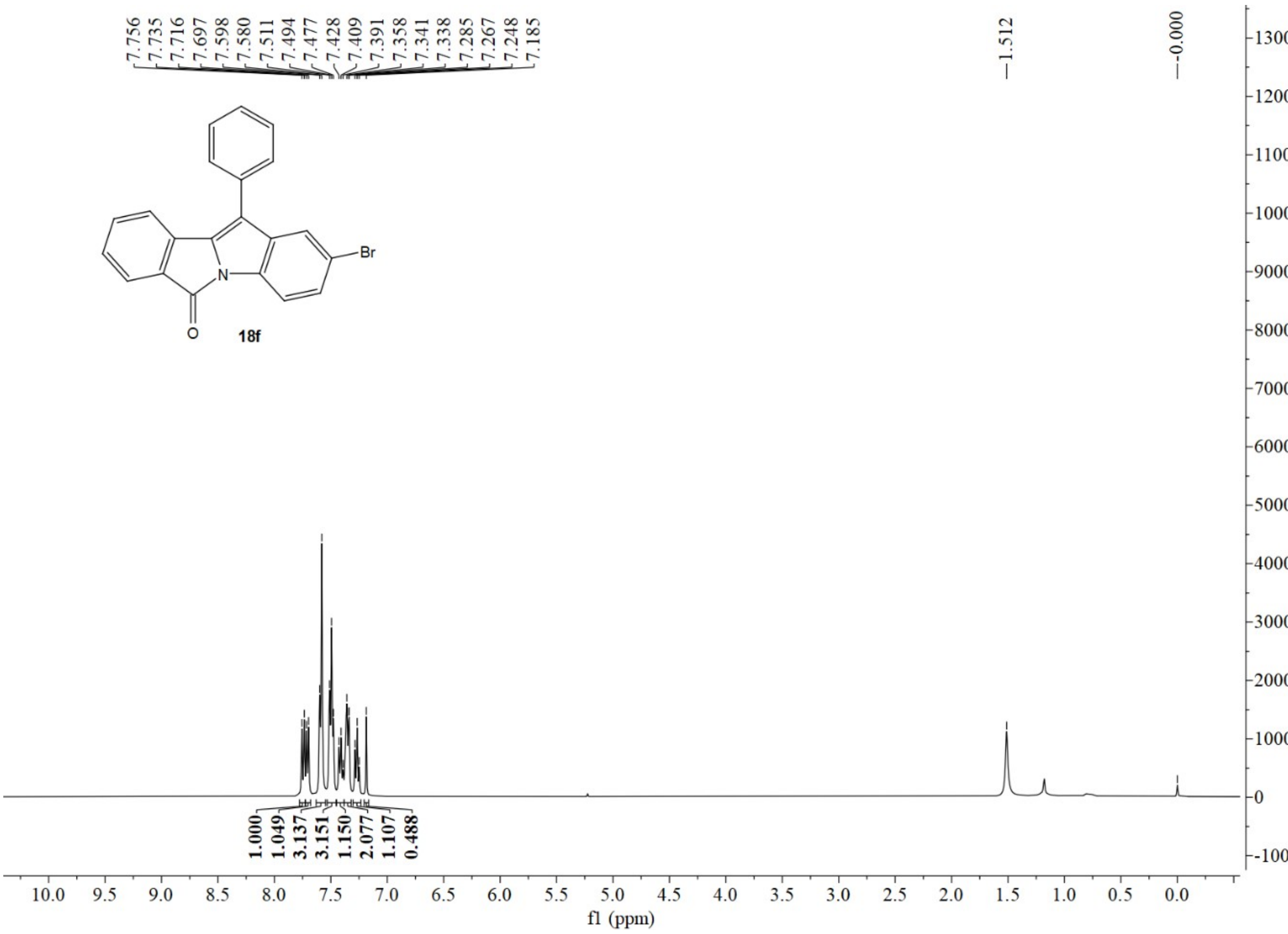

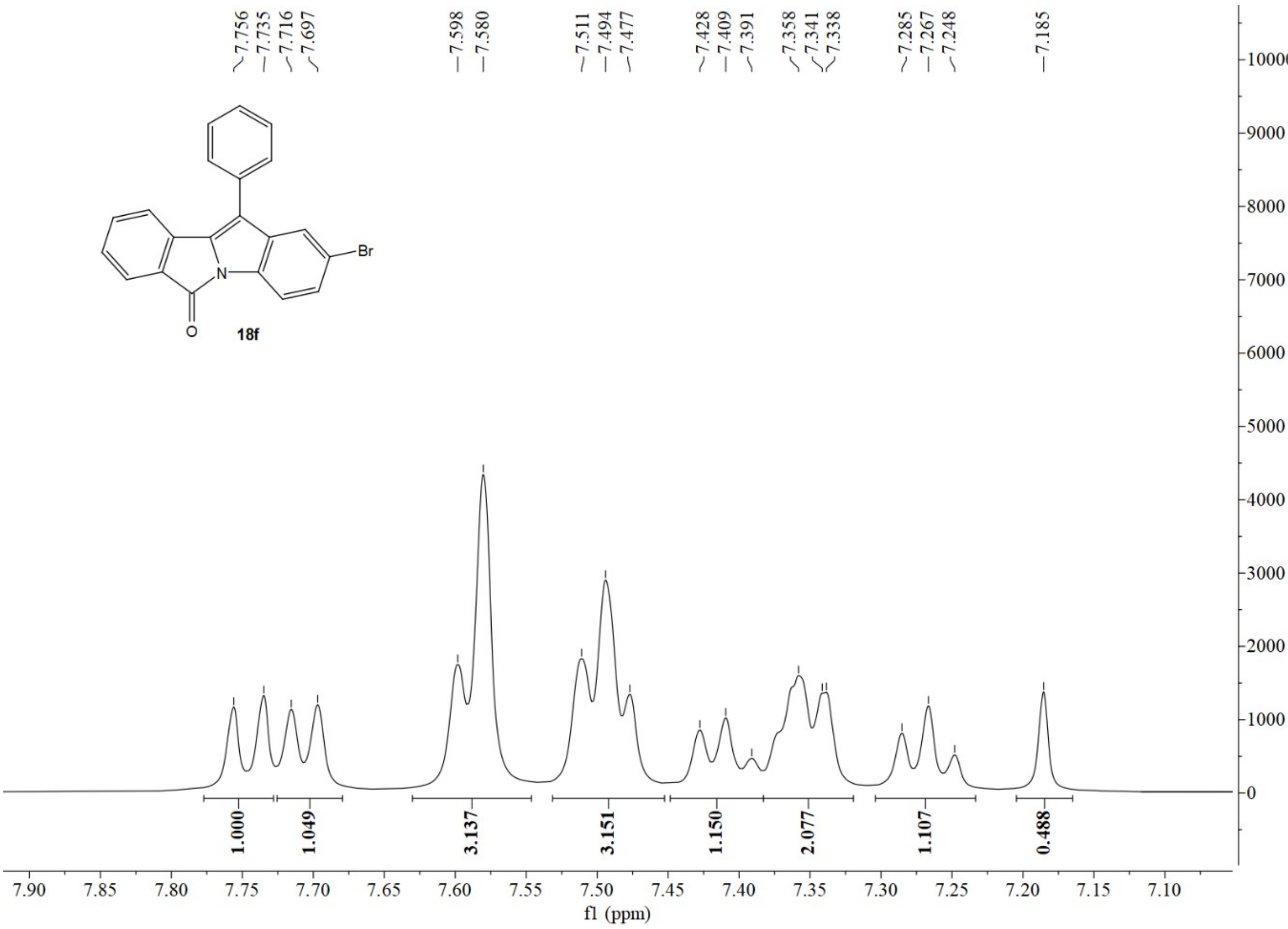

^{13}C NMR of 2-Bromo-11-phenyl-6H-isoindolo[2,1-a] indol-6-one (18f)

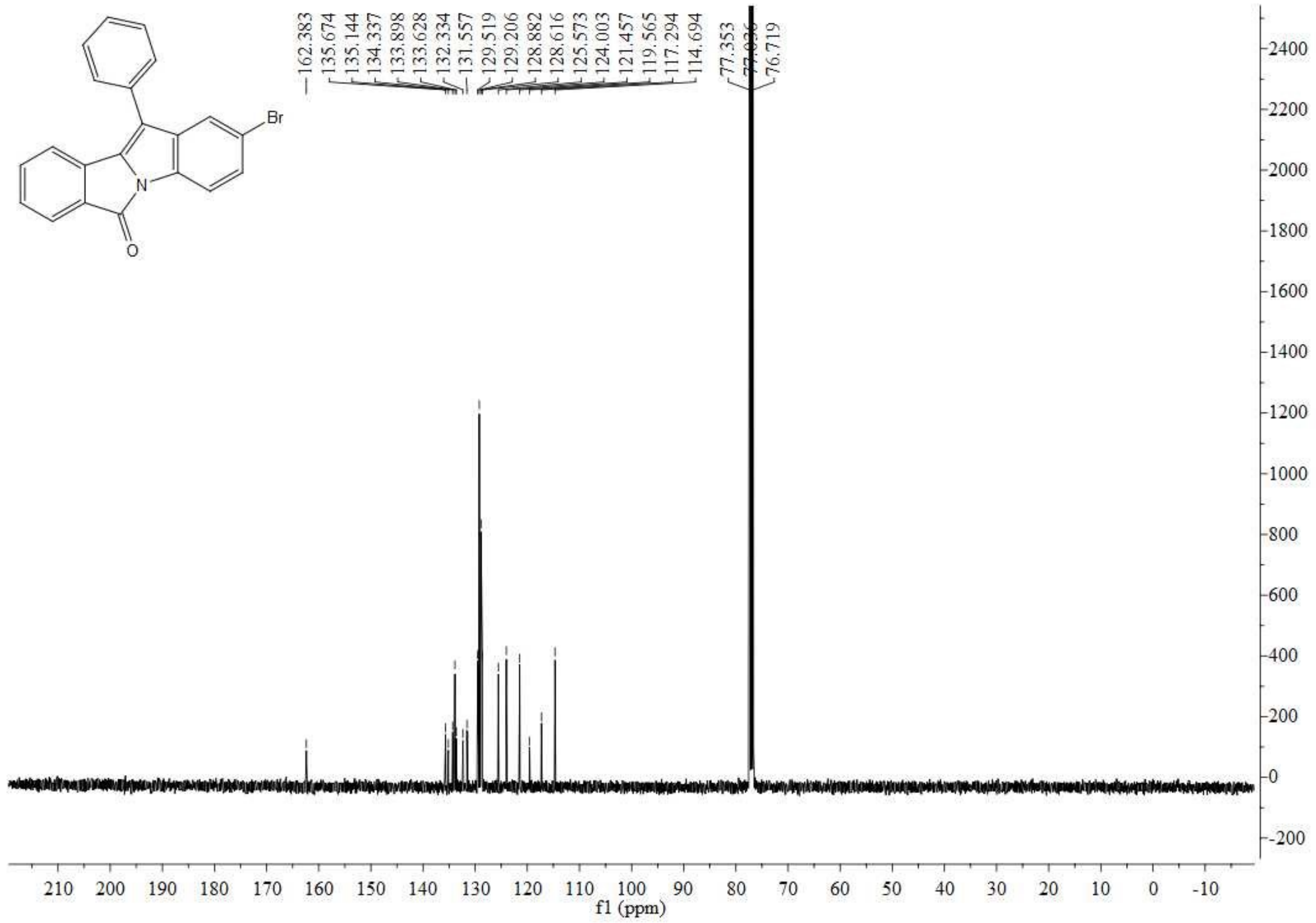

^{1}H NMR of 6-Oxo-11-phenyl-6H-isoindolo[2,1-a] indol-2-carbonitrile (18g)

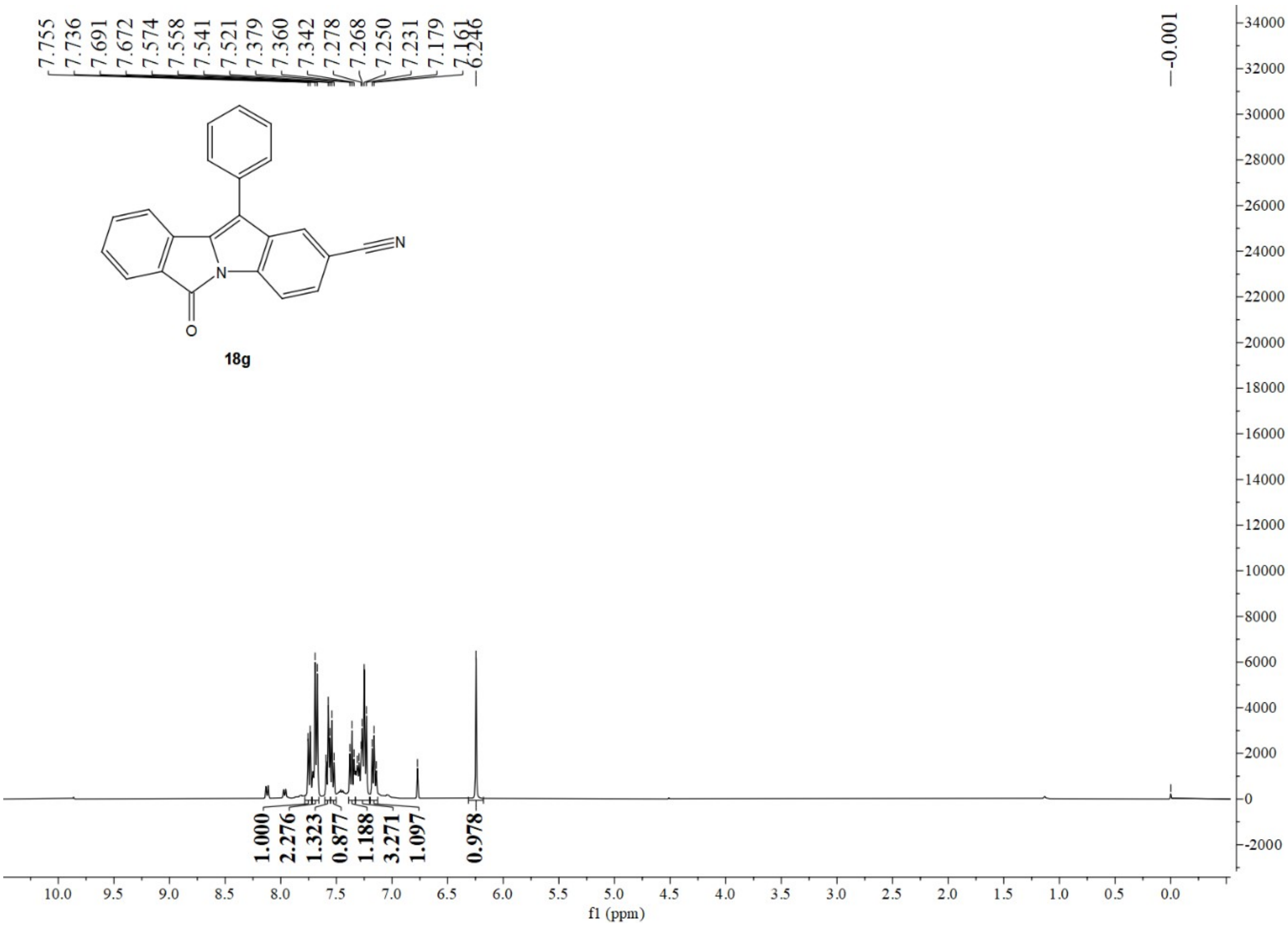

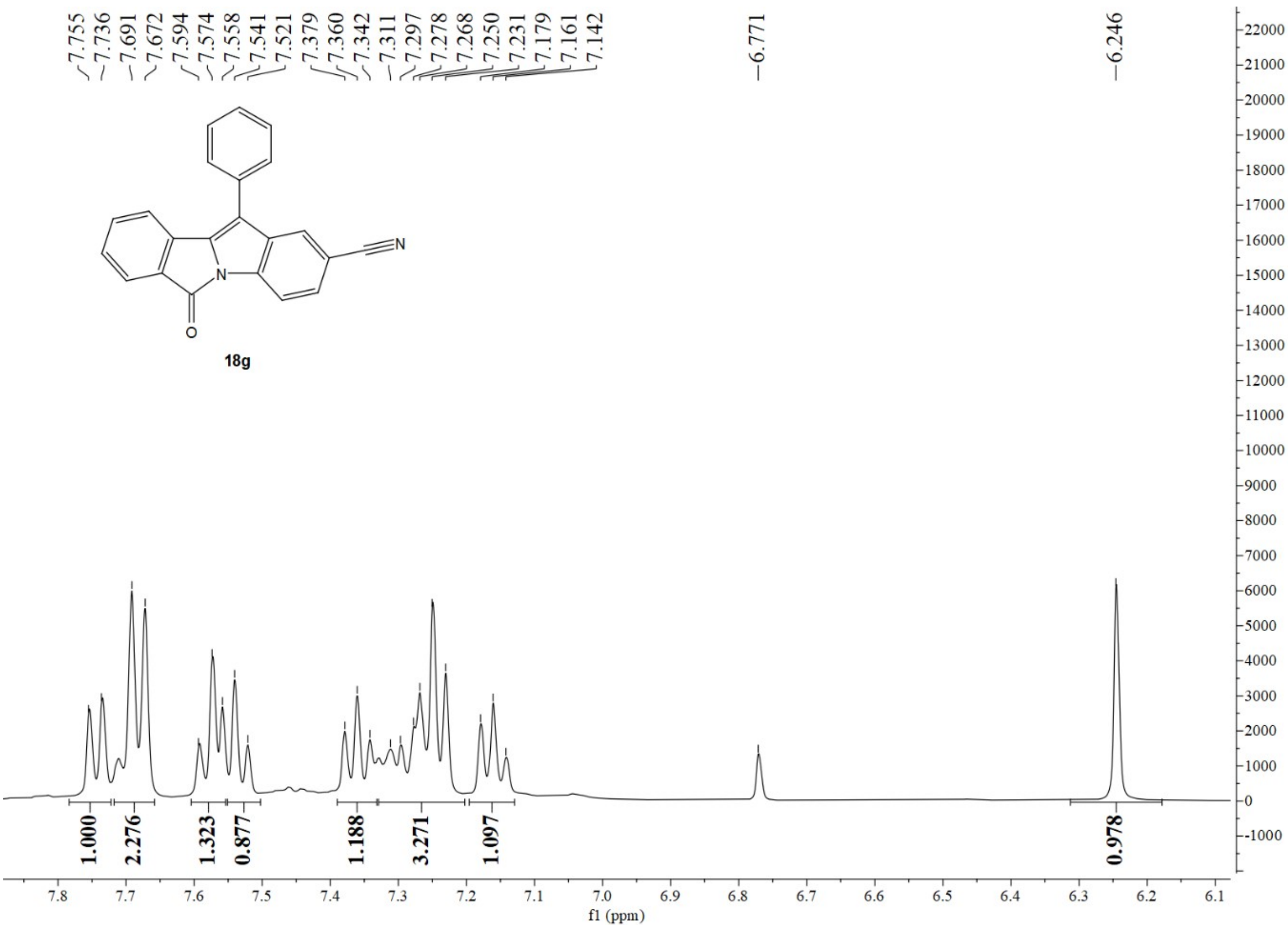

13C NMR of 6-Oxo-11-phenyl-6H-isoindolo[2,1-a] indol-2-carbonitrile (18g)

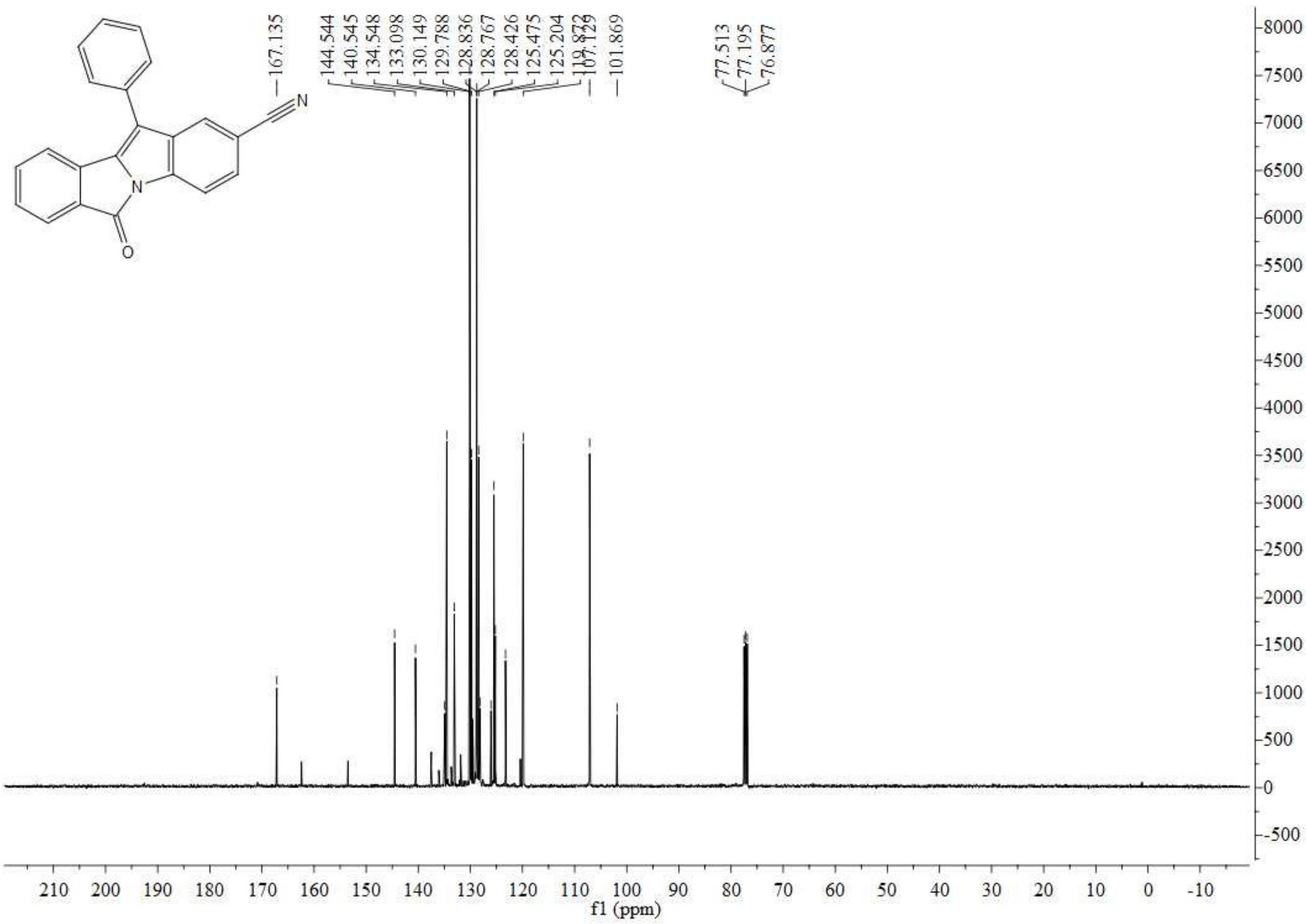

^{1}H NMR of 2-Methyl-11-phenyl-6H-isoindolo[2,1-a] indol-2-carbonitrile (18h)

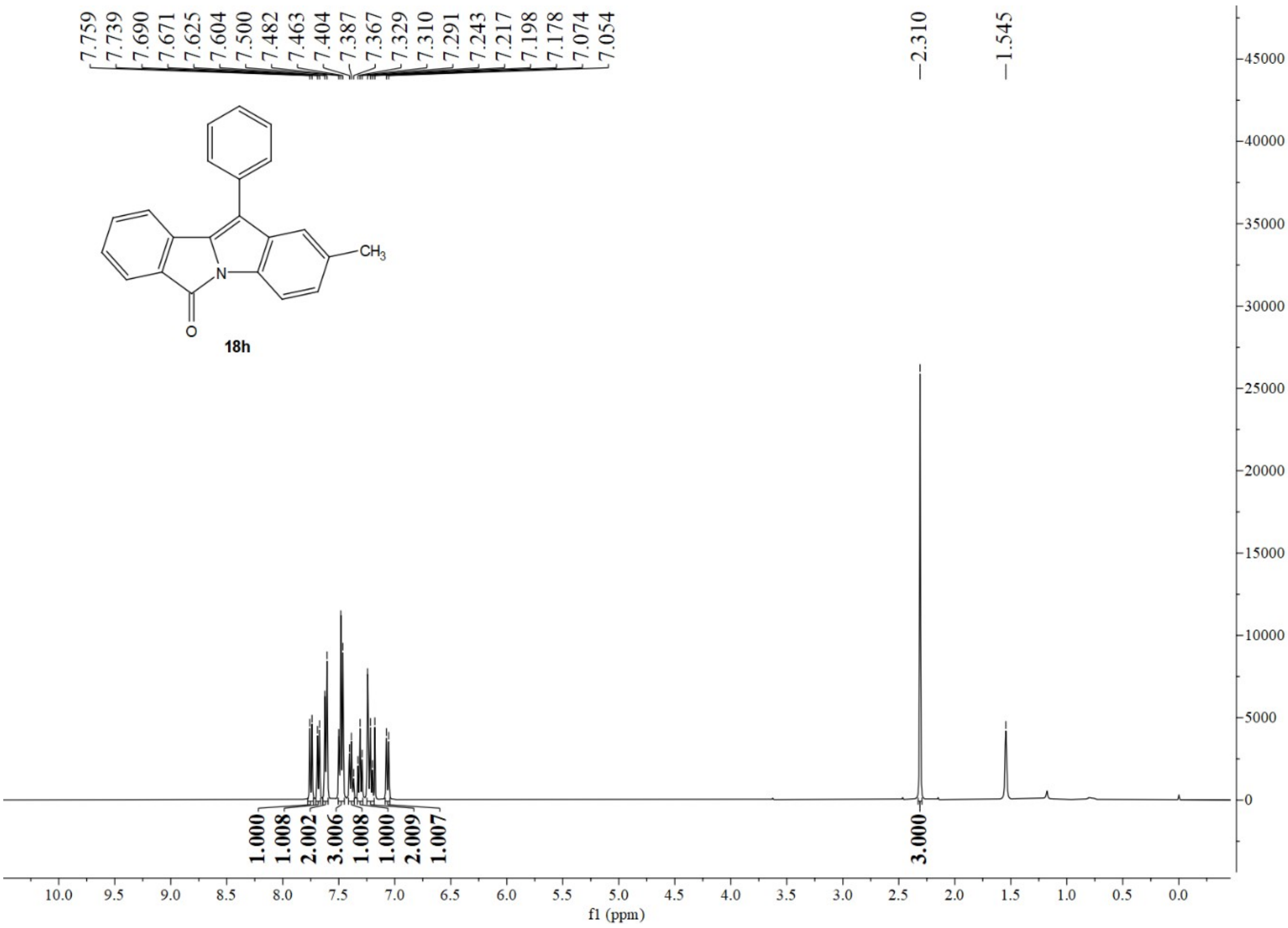

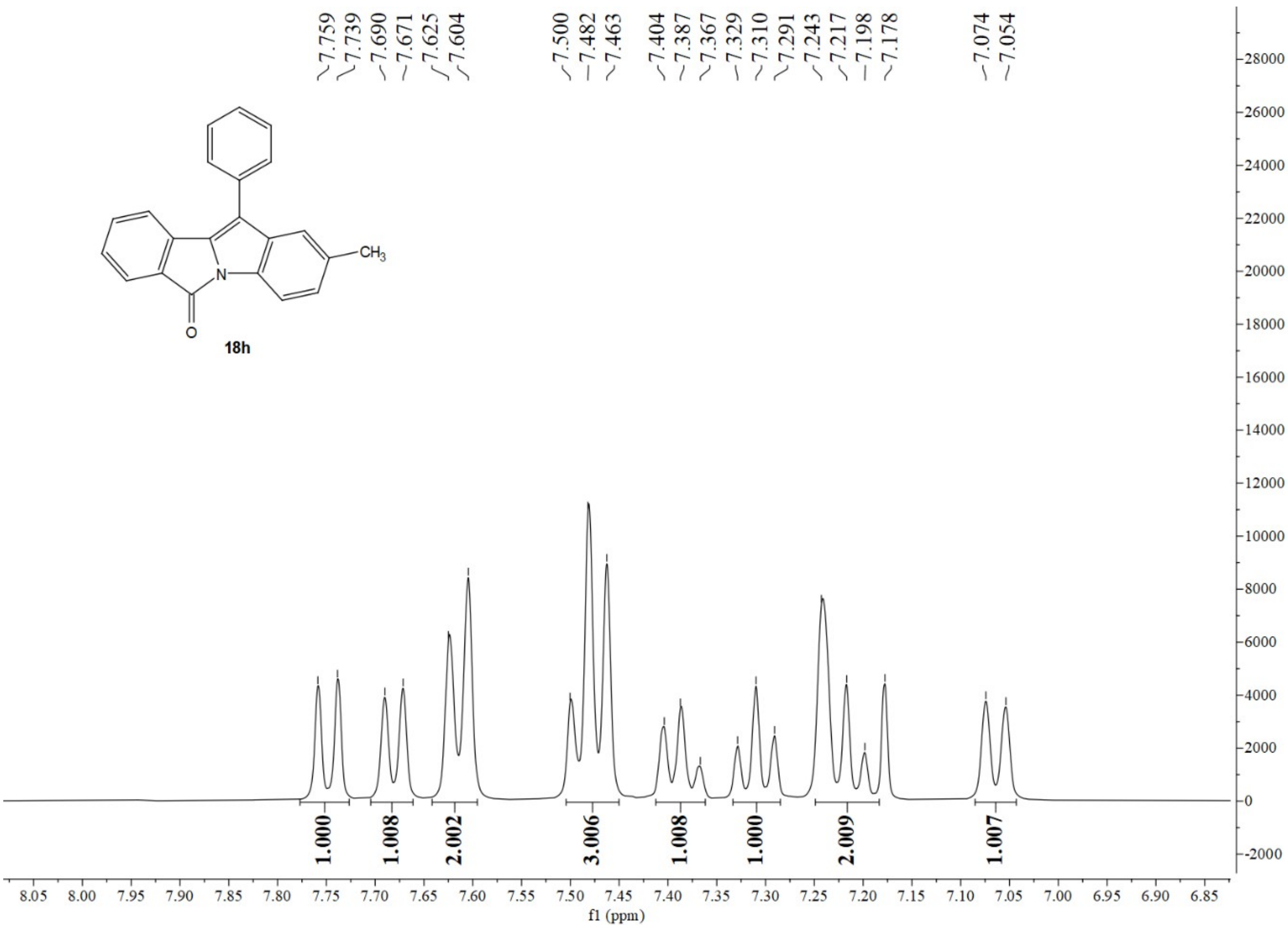

^{13}C NMR of 2-Methyl-11-phenyl-6H-isoindolo[2,1-a] indol-2-carbonitrile (18h)

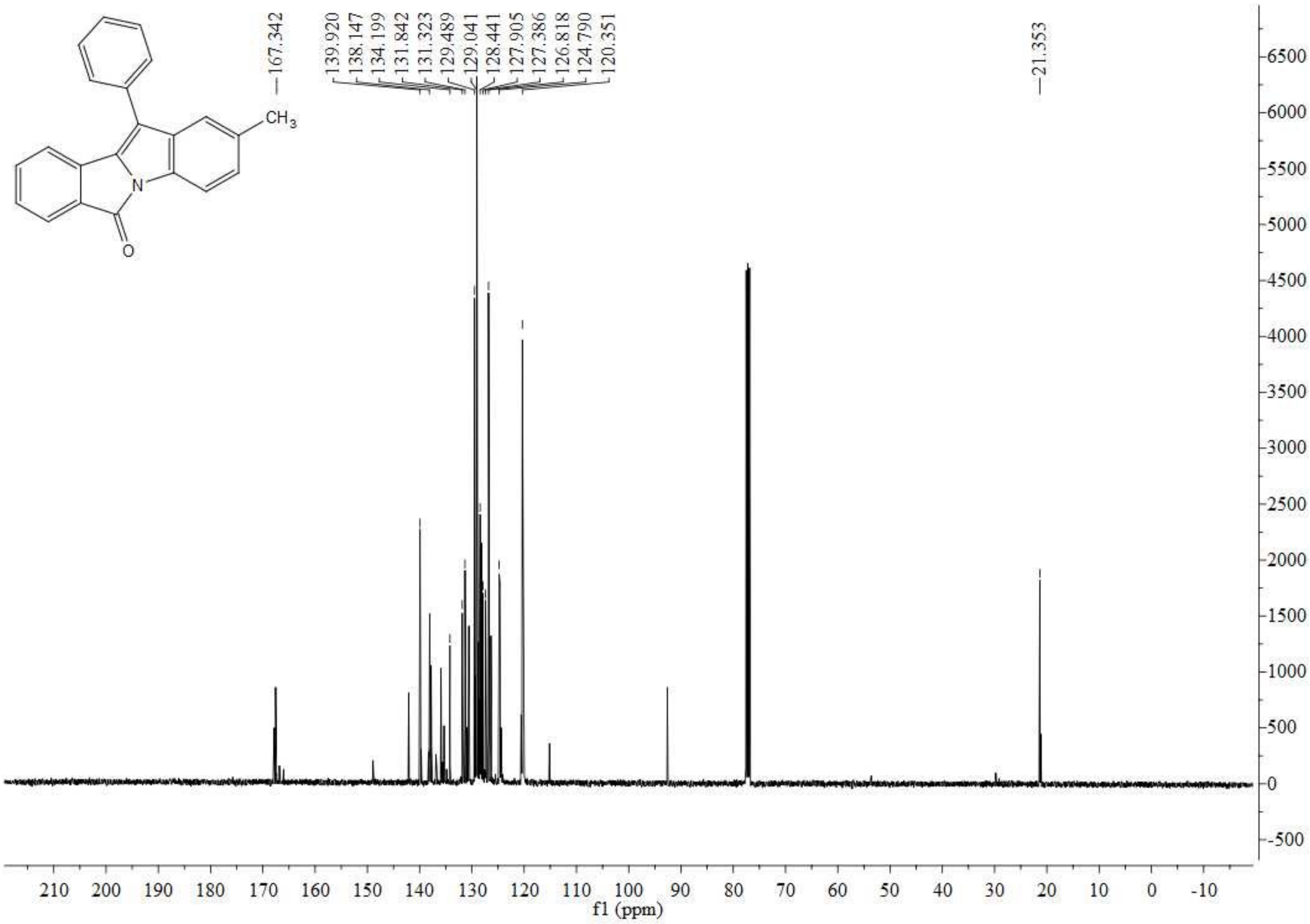

^{1}H NMR of 2-Methoxy-11-phenyl-6H-isoindolo[2,1-a] indol-2-carbonitrile (18i)

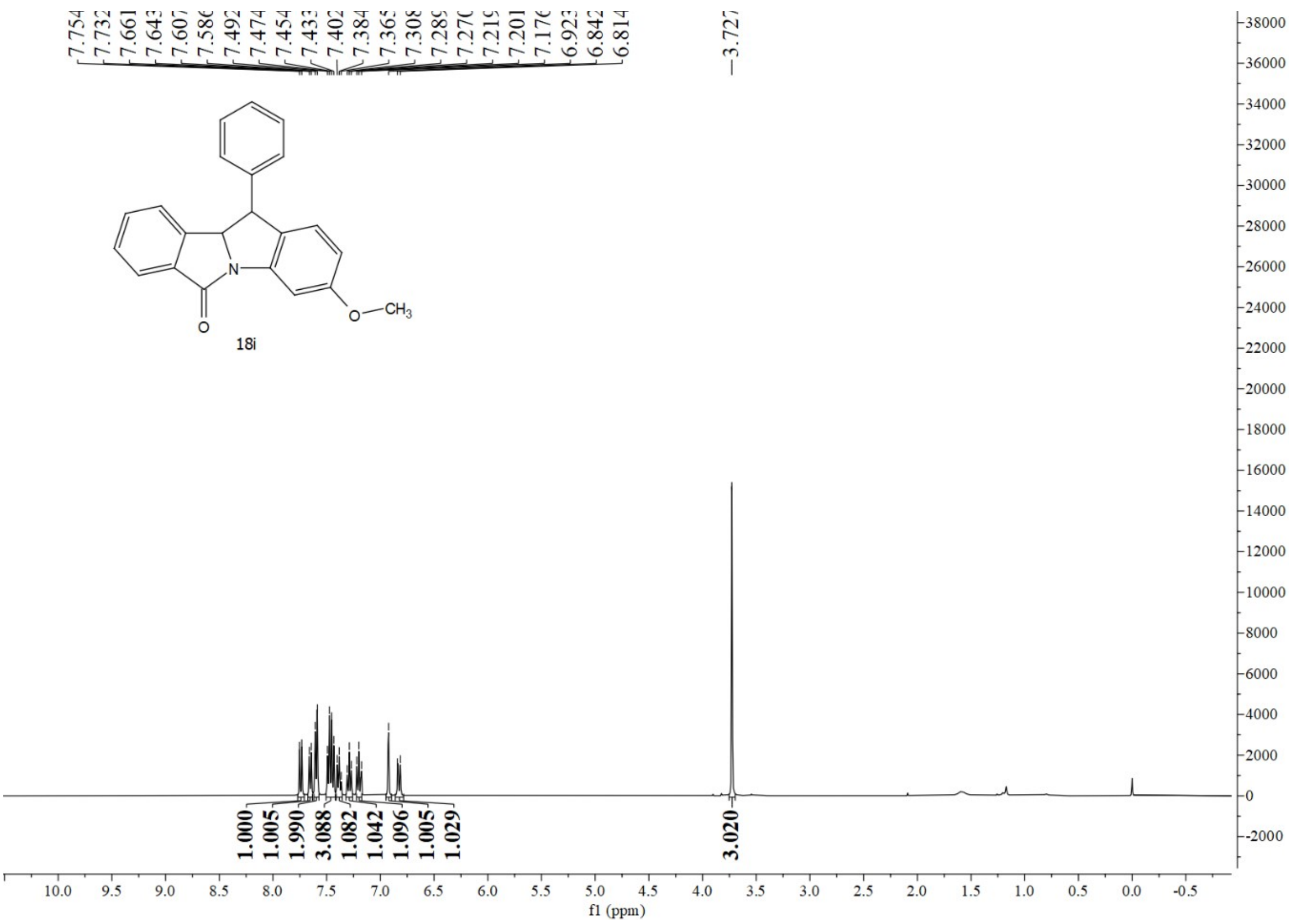

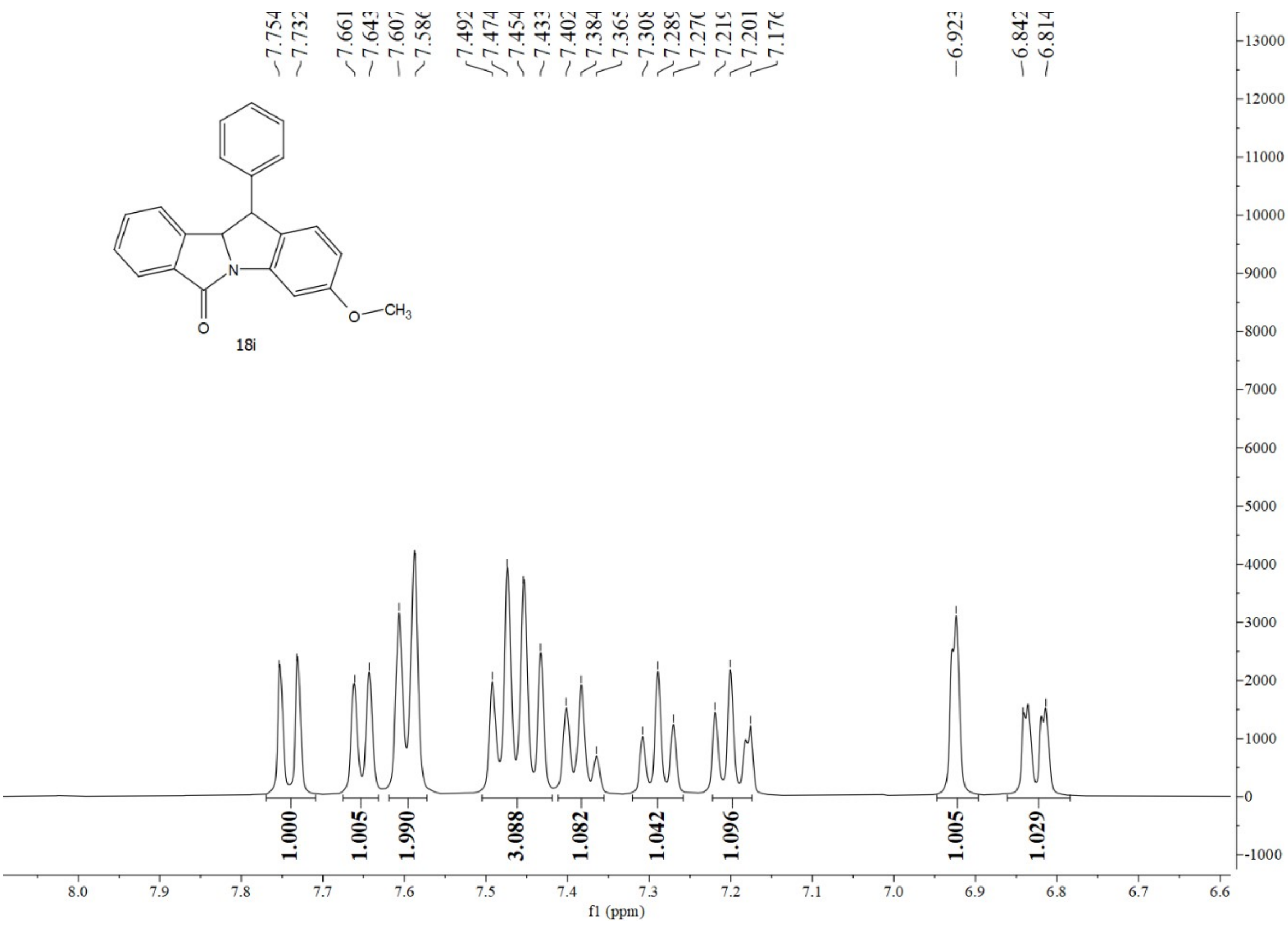

^{13}C NMR of 2-Methoxy-11-phenyl-6H-isoindolo[2,1-a] indol-2-carbonitrile (18i)

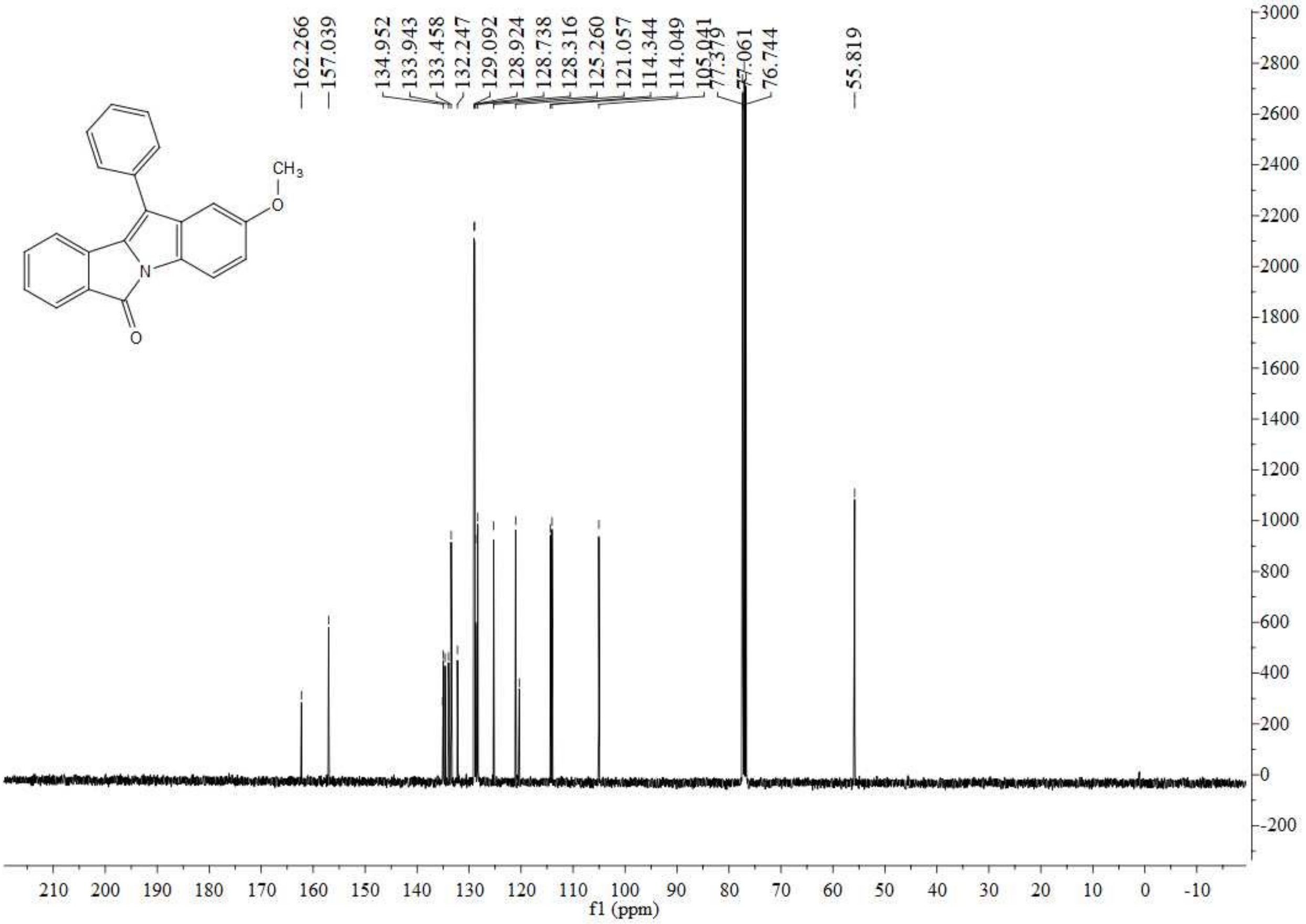

^{1}H NMR of 2-Butyl-11-phenyl-6H-isoindolo[2,1-a] indol-2-carbonitrile (18j)

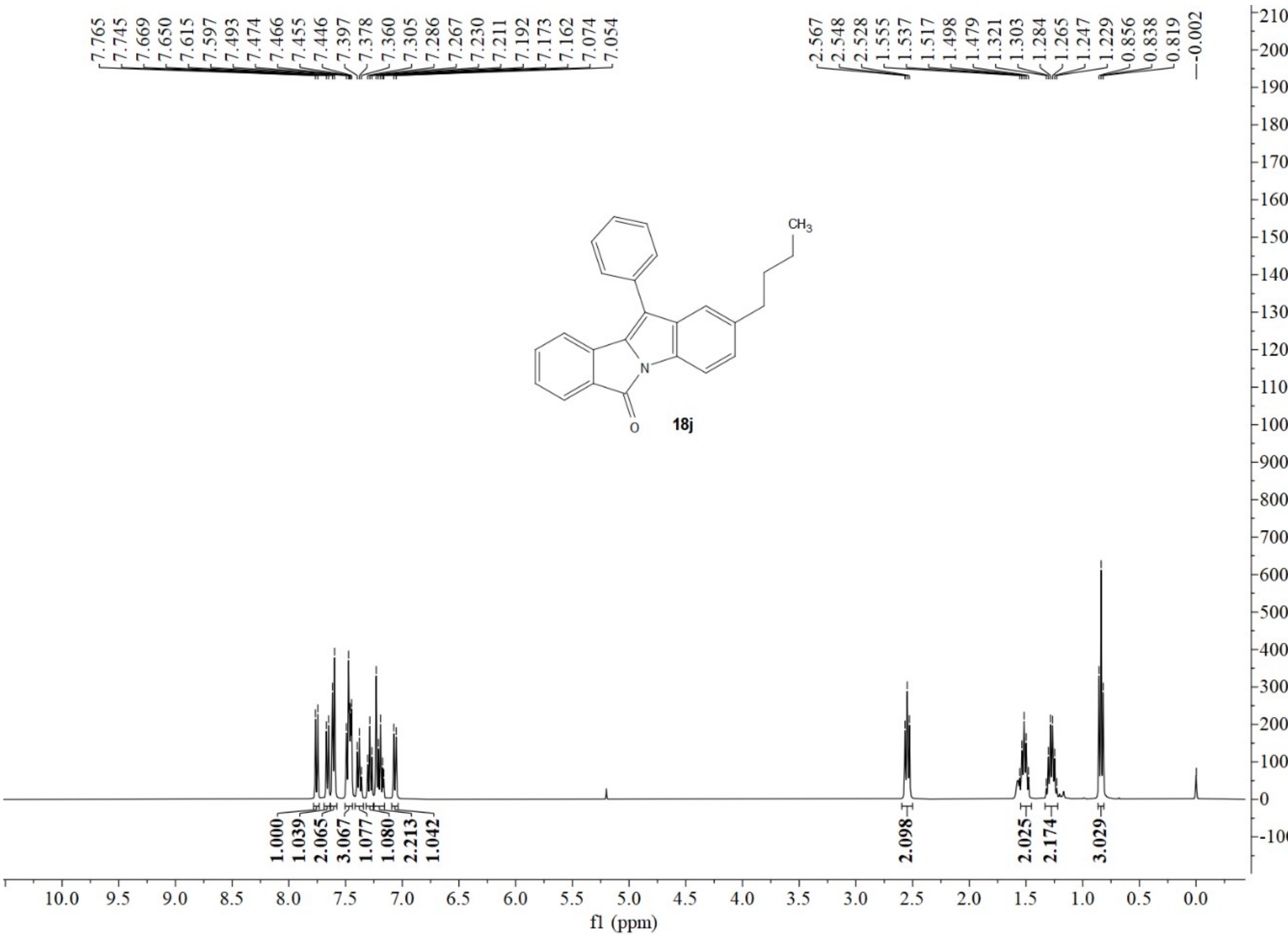

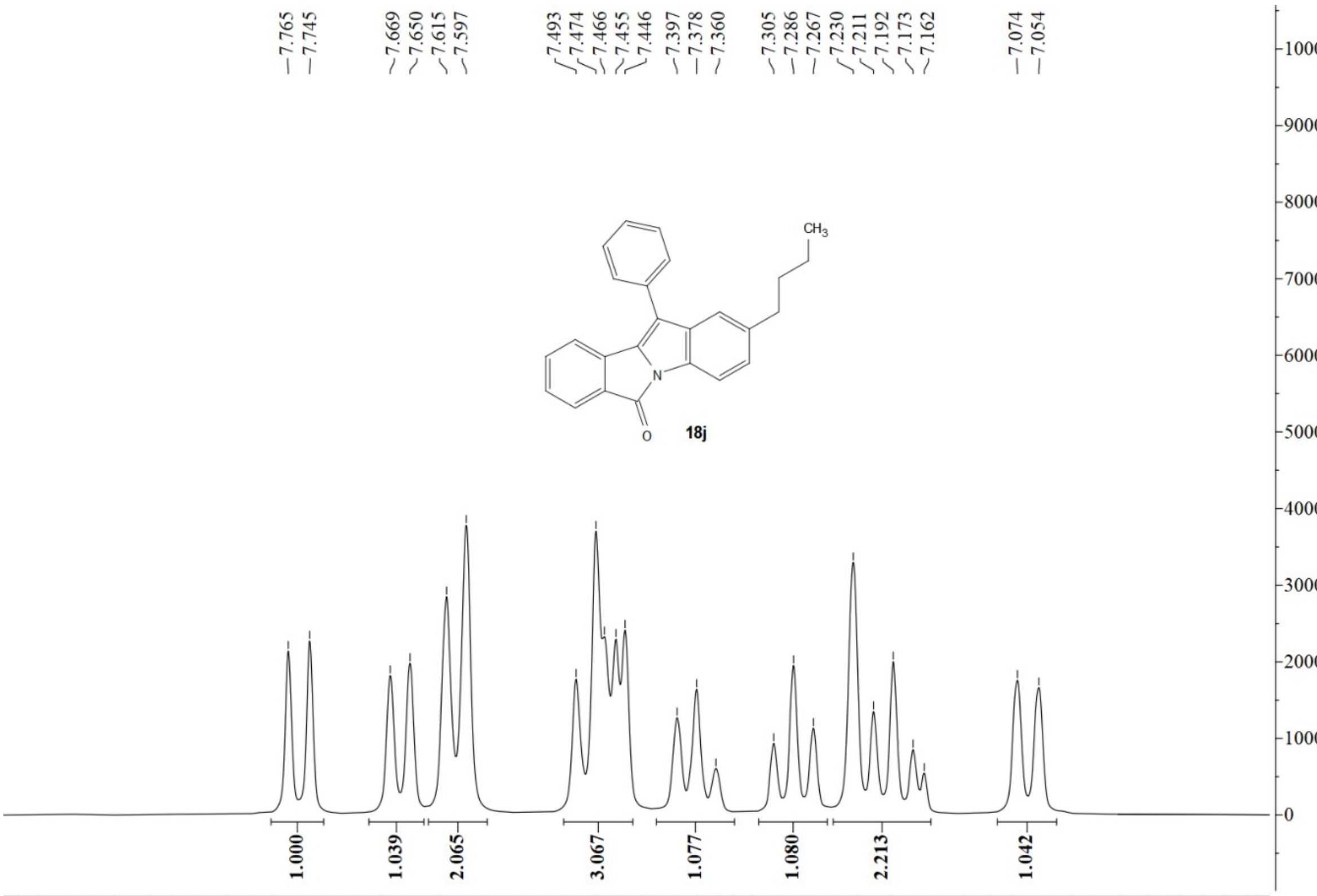

^{13}C NMR of 2-Butyl-11-phenyl-6H-isoindolo[2,1-a] indol-2-carbonitrile (18j)

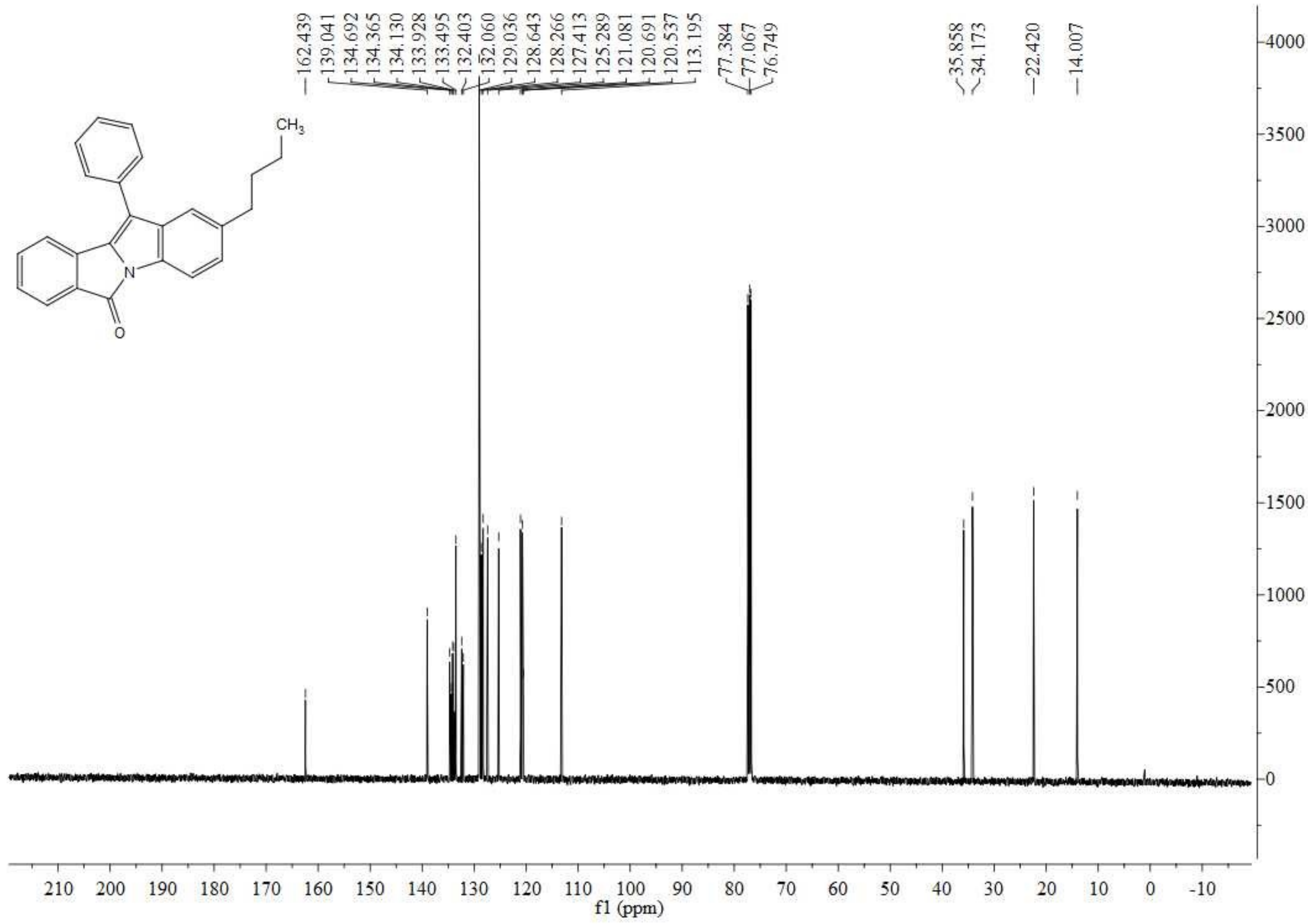

¹H NMR of 11-(4-butylphenyl)-2-methoxy-6H-isoindolo[2,1-a] indol-6-one (18k)

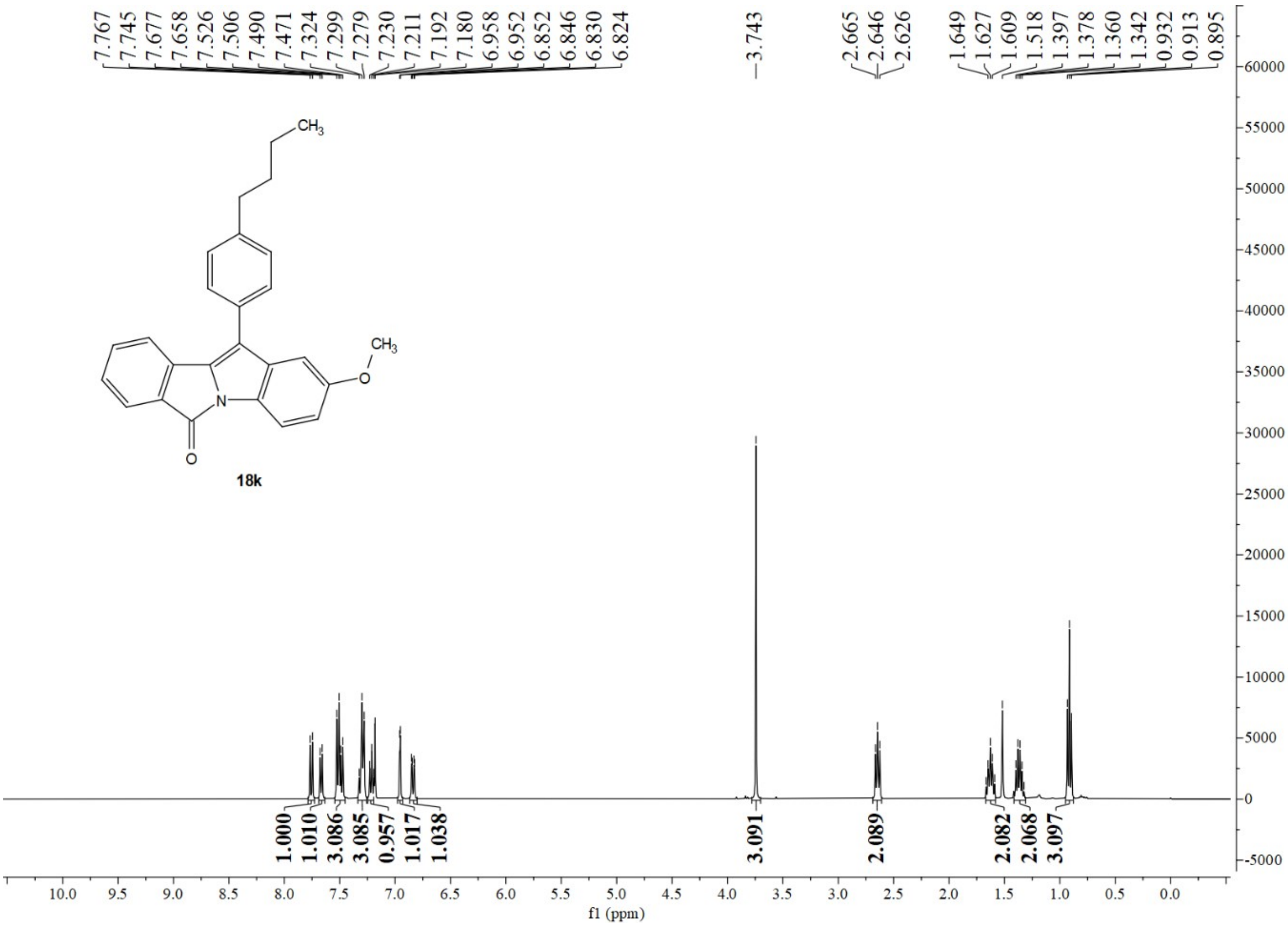

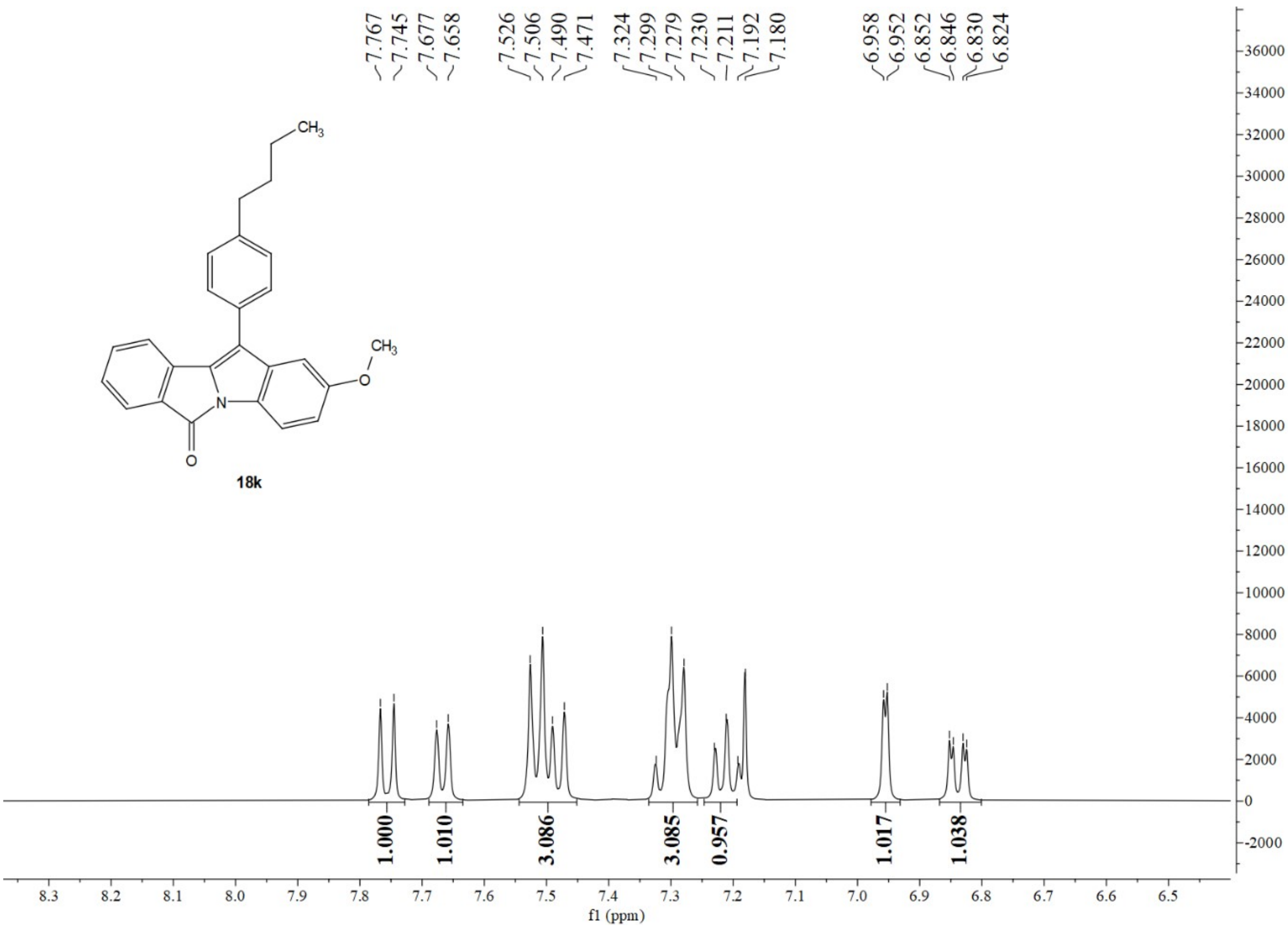

^{13}C NMR of 11-(4-butylphenyl)-2-methoxy-6H-isoindolo[2,1-a] indol-6-one (18k)

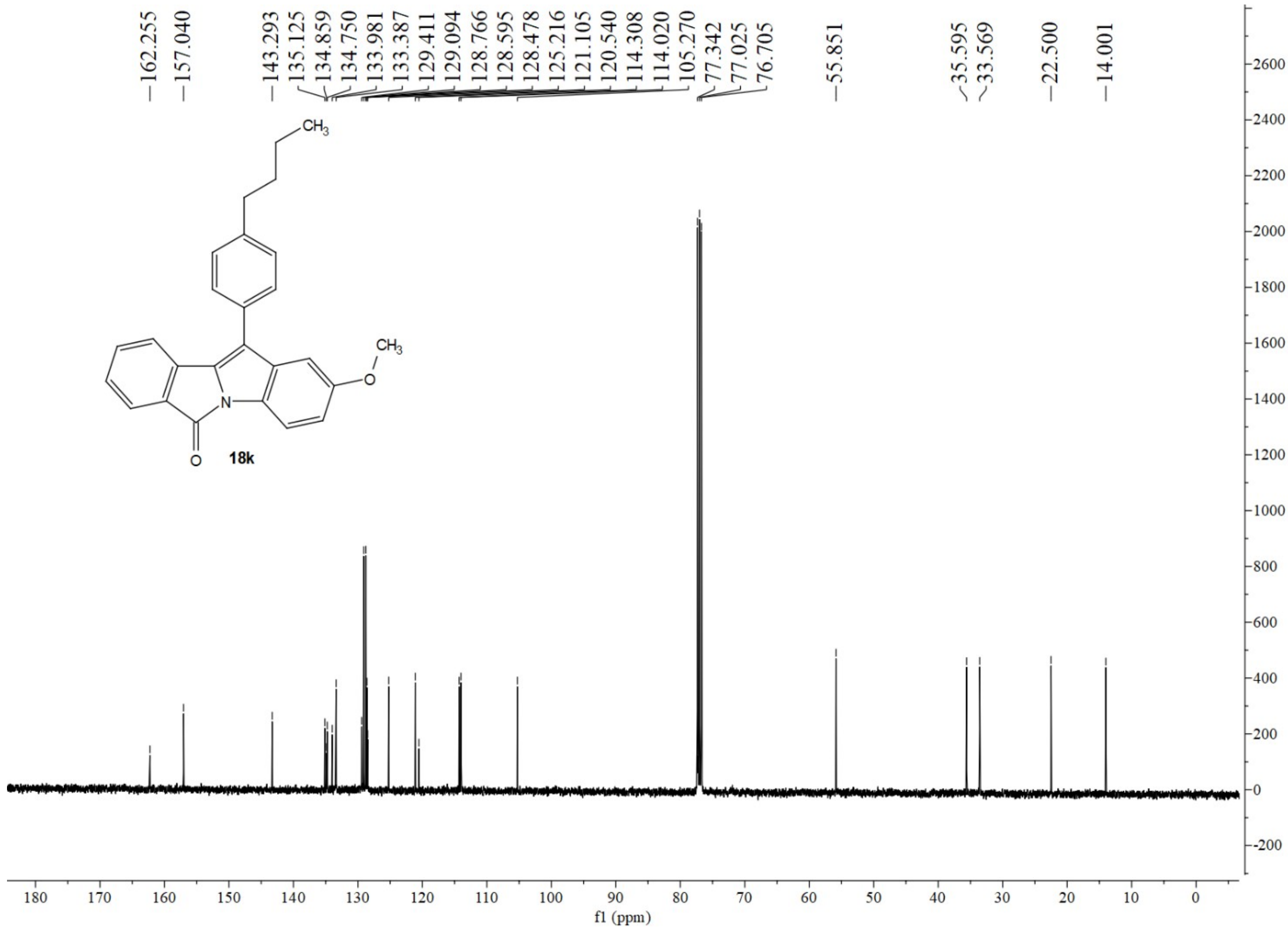

Supplement: Supplementary file 1 [file RA-016-D6RA05034D-s001.pdf]
